# Polymer/molecular semiconductor all-organic composites for high-temperature dielectric energy storage

**DOI:** 10.1038/s41467-020-17760-x

**Published:** 2020-08-06

**Authors:** Chao Yuan, Yao Zhou, Yujie Zhu, Jiajie Liang, Shaojie Wang, Simin Peng, Yushu Li, Sang Cheng, Mingcong Yang, Jun Hu, Bo Zhang, Rong Zeng, Jinliang He, Qi Li

**Affiliations:** State Key Laboratory of Power System, Department of Electrical Engineering, Tsinghua University, Beijing, China

**Keywords:** Nanocomposites, Energy storage, Electronic devices, Materials for energy and catalysis

## Abstract

Dielectric polymers for electrostatic energy storage suffer from low energy density and poor efficiency at elevated temperatures, which constrains their use in the harsh-environment electronic devices, circuits, and systems. Although incorporating insulating, inorganic nanostructures into dielectric polymers promotes the temperature capability, scalable fabrication of high-quality nanocomposite films remains a formidable challenge. Here, we report an all-organic composite comprising dielectric polymers blended with high-electron-affinity molecular semiconductors that exhibits concurrent high energy density (3.0 J cm^−3^) and high discharge efficiency (90%) up to 200 °C, far outperforming the existing dielectric polymers and polymer nanocomposites. We demonstrate that molecular semiconductors immobilize free electrons via strong electrostatic attraction and impede electric charge injection and transport in dielectric polymers, which leads to the substantial performance improvements. The all-organic composites can be fabricated into large-area and high-quality films with uniform dielectric and capacitive performance, which is crucially important for their successful commercialization and practical application in high-temperature electronics and energy storage devices.

## Abstract (web summary)

Dielectric polymers are widely used in electrostatic energy storage but suffer from low energy density and efficiency at elevated temperatures. Here, the authors show that all-organic composites containing high-electron-affinity molecular semiconductors exhibit excellent capacitive performance at 200 °C.

## Introduction

Dielectric polymers are widely used in electrostatic capacitors for the well-recognized advantages such as high-voltage endurance, low energy loss and great reliability^1,2^. The building up of extreme-environment electronic devices, circuits, and systems entails high temperature-capable electronic materials, among which dielectric polymers for high-voltage capacitors are becoming the bottleneck^3–5^. The high temperature operation of dielectric polymers is limited by a fundamental issue that the thermally and electrically assisted charge injection, excitation and transport can lead to exponential increase of leakage current, and hence low discharged energy density (*U*_e_) and poor discharge efficiency (*η*, defined as the ratio of discharged to stored energy densities)^6–8^. Therefore, despite the numerous synthetic and modification approaches to high-temperature dielectric polymers^9–13^, appreciable *U*_e_ (>2.0 J cm^−3^) remains accessible only below 150 °C and is usually accompanied with low *η* that would cause massive waste heat (Joule heating) and even thermal runaway of the device^14^.

Recent studies demonstrate that insulating, inorganic nanostructures (e.g., boron nitride nanosheets) can be utilized to impede the transport of charge carriers in dielectric polymers^14–18^. The resultant polymer nanocomposites show improved capacitive performance at elevated temperatures, achieving concurrent high *U*_e_ (varying from 2.2 to 4.8 J cm^−3^) and high *η* (~90%) up to 150 °C. Nevertheless, as the temperature increases further, both the energy density and efficiency of the nanocomposites decline abruptly. At 200 °C, the maximum *U*_e_ above 90% *η* is only 0.5–1.3 J cm^−3^, which is far below the room-temperature energy density (~4.0 J cm^−3^) of the commercial benchmark capacitor dielectric biaxially oriented polypropylene (BOPP)^1^. Although higher *U*_e_ can be achieved at the expense of a much reduced *η*, the significant heat generation could cause damage to the device. Such performance falls well short of the requirement for conversion and control of electrical energy in many of the emerging applications ranging from distributed power converters in electrified aircraft to electrical compressors in deep oil and gas extraction^3–5,7^, where the operating temperature can reach or exceed 200 °C. Moreover, the insulating nanostructures^14–17^ are synthesized in low yields (e.g., nanosheets and nanowires), have high surface energies (i.e., unsatisfactory compatibility with polymers), and are loaded at high concentrations (c.a. 10 vol.%), rendering the manufacture of large-area and uniform nanocomposite films challenging^19^.

**Fig. 1:**
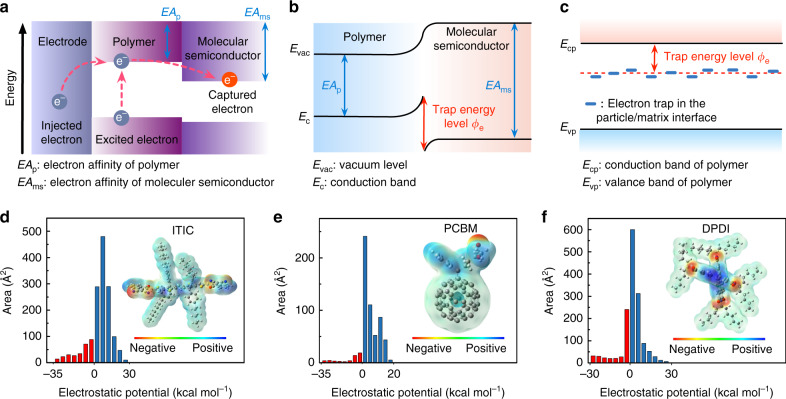
Design of the dielectric polymer/molecular semiconductor all-organic composite. **a** Band diagram showing the possible charge transfer in the all-organic composite. **b** Schematic illustration of trap energy level introduced by the molecular semiconductors in the all-organic composite. The trap energy level can be calculated using *Φ*_e_ = EA_ms_ − EA_p_, where EA_ms_ and EA_p_ are the electron affinities of the molecular semiconductor and the dielectric polymer, respectively. **c** Schematic representation of the trap energy level in polymer/insulating particle nanocomposites introduced through modification of polymer chain conformation and arrangement in the particle/matrix interfaces. The electron traps are located in the forbidden gap and close to the conduction band of the dielectric polymer. **d**–**f** Electrostatic potential distribution and area percentage in each electrostatic potential range of ITIC, PCBM, and DPDI.

In this work, we depart from the previous approaches and show that a scalable all-organic composite comprising dielectric polymers blended with a low concentration (0.25–0.75 vol.%) of high-electron-affinity molecular semiconductors can attain record energy storage performance up to 200 °C. The rationale of our material design (Fig. 1a) is that the molecular semiconductors possessing significantly higher electron affinities (EA_ms_, e.g., 4.2 eV) than those of the dielectric polymers (EA_p_, e.g., 2.7 eV, Supplementary Figs. 1–4) can capture the injected and excited electrons via strong electrostatic attraction. This would give rise to a large trap energy level (*Φ*_e_ = EA_ms_ − EA_p_, e.g., 1.5 eV) for the captured electrons to escape from the trap sites (Fig. 1b). This is fundamentally different from the insulating nanostructures in the previous high-temperature polymer nanocomposites, which introduce trap sites mainly through modification of the polymer chain conformation and arrangement in the particle/matrix interface (the *Φ*_e_ is usually below 1.0 eV) (Fig. 1c)^14,20,21^. We hypothesize that the deep traps introduced by the molecular semiconductors would firmly immobilize the free electrons in the polymer even under high temperature and high electric field conditions if the molecular semiconductors are thermally stable and their concentrations are well below the percolation threshold. And these deep traps are anticipated to affect both the charge injection and transport.

## Results

### Composition and electrical resistivity

To verify our hypothesis, we selected three commercial molecular semiconductors, i.e., ITIC, PCBM, and DPDI (the full names can be found in “Methods”) with the electron affinities of 3.9, 4.2 and 4.0 eV, respectively^22–24^. These molecular species are thermally stable below 300 °C (refs. ^25–27^), and are commonly used in organic solar cells based on donor-acceptor blends as they have highly electronegative elements and provide strong electron-accepting abilities^23,28^. The electronegative elements in these molecules are displayed in the density functional theory (DFT) computation of the electrostatic potential distribution (Fig. 1d–f). The quantitative molecular surface analysis reveals more positive electrostatic potential on their surfaces, suggesting the attractive nature to electrons. The thermally stimulated depolarization current (TSDC) measurement confirms that blending the molecular semiconductors into a typical heat-resistant dielectric polymer (PEI, i.e., polyetherimide) brings extra carrier trap sites in the resultant all-organic composites, with the trap energy level estimated to be around 1.5 eV (Supplementary Fig. 5). We found that the all-organic composites exhibited significantly higher electrical resistivity with respect to the pristine polymer at the optimal compositions (0.25, 0.50 and 0.75 vol.% of the semiconductors for the PEI/ITIC, PEI/PCBM and PEI/DPDI composites, respectively) (Supplementary Figs. 6 and 7). For example, under an electric field of 200 MV m^−1^ and 200 °C, the electrical resistivity of the PEI/PCBM is 8.54 × 10^12^ Ω m, over two orders of magnitude higher than that of the pristine PEI (7.82 × 10^10^ Ω m). This is unexpected from the knowledge that the molecular semiconductors have higher electrical conductivity than the dielectric polymer, and that they have marginal impact on the dielectric constant, dissipation factor (Supplementary Fig. 8) and thermal stability (Supplementary Fig. 9) of the composite. The Fourier-transform infrared (FTIR) investigation (Supplementary Fig. 10) suggests that there is no significant interaction between the dielectric polymers and the molecular semiconductors after mixing.

### Charge injection and transport

**Fig. 2:**
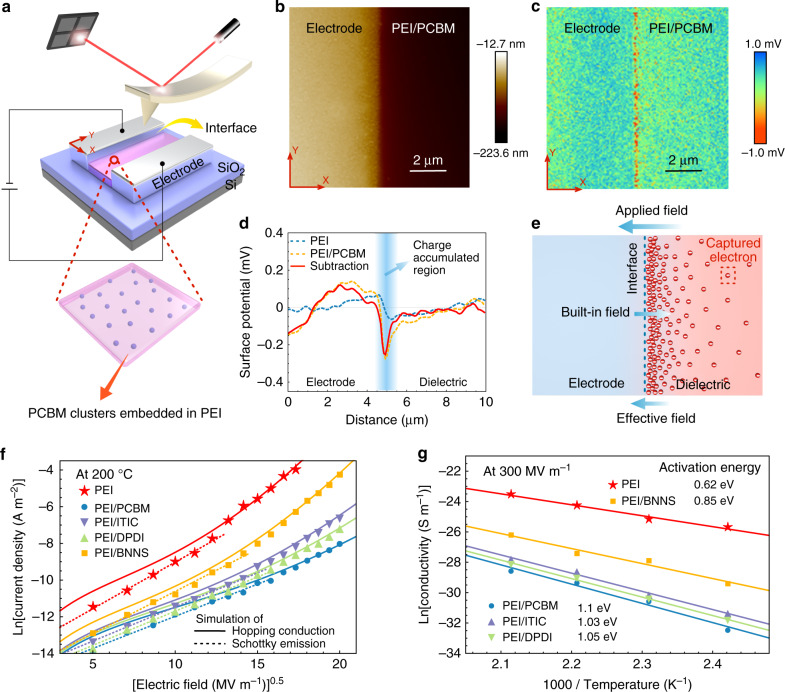
Mechanism to the suppression of electrical conduction. **a** Schematic diagram of the KPFM testing. The circuit is used for charge injection prior to the measurement, and is disconnected during the measurement. **b** Topography of the PEI/PCBM composite. **c** Surface potential mapping of the PEI/PCBM composite. **d** Surface potential profiles along the horizontal axis derived from the surface potential mappings (**c** and Supplementary Fig. 14b). The amplitude of surface potential is averaged along the *y* direction in the surface potential mappings. The subtraction (red curve) of surface potential between the pristine PEI and the PEI/PCBM composite excludes the influence of the different energy levels between the electrode and the polymer. **e** Schematic illustration of the built-in electric field formed by the molecular semiconductor captured electrons. **f** Current density as a function of applied electric field of the pristine PEI, the all-organic composites and the PEI/BNNS nanocomposite measured at 200 °C. **g** Arrhenius function of conductivity for the pristine PEI, the all-organic composites and the PEI/BNNS nanocomposite measured at the applied electric field of 300 MV m^−1^.

To demonstrate that the exceptional electrical property of the all-organic composites results from the effect of the molecular semiconductors on the charge injection and transport, we first carried out a spatially resolved detection of surface potential using Kelvin probe force microscopy (KPFM) to gauge the injected charges (Fig. 2a, Supplementary Fig. 11 and Supplementary Note 1). The all-organic composite sample PEI/PCBM was cast between a pair of metal electrodes with the electrode/dielectric interface clearly exposed (Fig. 2b, and see “Methods” for details). The PCBM tends to form ultrafine clusters of a uniform diameter (~2 nm)^29^ that are found to be dispersed homogeneously in the polymer matrix (Supplementary Fig. 12). Charge injection was implemented prior to the KPFM measurement using an external circuit, which was then disconnected during the scan (Supplementary Fig. 13 and Supplementary Note 2). Interestingly, when scanning across the cathode edge of the PEI/PCBM sample, there is a distinct line signal of negative surface potential along the *y* direction at the dielectric side (Fig. 2c). The average amplitude of surface potential plotted as a function of the distance to the cathode edge in the *x* direction (Fig. 2d) reveals a negative peak value in the vicinity of the electrode/dielectric interface. Since this line signal is absent either in the pristine PEI or in the PEI/PCBM without implementing the charge injection (Supplementary Fig. 14), it is attributed to the injected electrons captured by the PCBMs. The steeply decreased amount of electrons (i.e., increased surface potential) along the *x* direction toward the dielectric side suggests that the immobilized electrons form a charge-accumulated region near the electrode/dielectric interface (the width is below 800 nm) and establish a built-in electric field in the opposite direction to the applied field, which suppresses further net inflow of electrons (Fig. 2e). The pulsed electro-acoustic (PEA) measurement at an applied electric field of 200 MV m^−1^ and 100 °C confirms that such built-in field repels the charge injection (Supplementary Figs. 15 and 16).

Charge transport behavior was learnt through analysis of the conduction current as a function of the applied electric field and temperature. The fitting^30^ of current density versus field measured at 200 °C implies that a transition from charge injection-governed conduction (Schottky emission) to the transport-limited hopping conduction occurs in the pristine PEI as the field strength increases from 25 to 300 MV m^−1^ (Fig. 2f). The all-organic composites display very similar pattern to the pristine PEI in the fitting curves but the fittings reveal unmatched dielectric constants necessary for validating the typical Schottky emission model in the lower electric field regime (Supplementary Note 3). We speculate that the presence of the built-in field restricting the charge injection leads to the modified conduction mechanism in the lower electric field regime because other plausible conduction mechanisms are excluded (Supplementary Fig. 17). In the higher electric field regime, the transition to transport-limited conduction in the all-organic composites is most likely due to the saturation of charge trapping near the electrode/dielectric interface (hence the saturation of the built-in field strength). The fittings in the higher electric field regime suggest that the all-organic composites possess greater population density of the trap sites (i.e., shorter mean spacing between trap sites, *λ*, 0.89–1.24 nm) than both the pristine PEI (*λ* = 1.84 nm) and a nanocomposite comprising PEI and 10 vol.% of boron nitride nanosheets (PEI/BNNS, *λ* = 1.67 nm) (Supplementary Note 4). The activation energies associated with the carrier trapping are determined from the results of temperature-dependent electrical conductivity (Fig. 2g and Supplementary Note 5). Clearly, the all-organic composites have higher activation energies than the PEI/BNNS, indicative of a greater trap depth^14^. These results in conjunction with the KPFM study corroborate that the molecular semiconductors have major impacts on the charge injection and transport in dielectric polymers, which are more effective than the insulating nanostructures in immobilizing the free charges.

### Capacitive energy storage performance

**Fig. 3:**
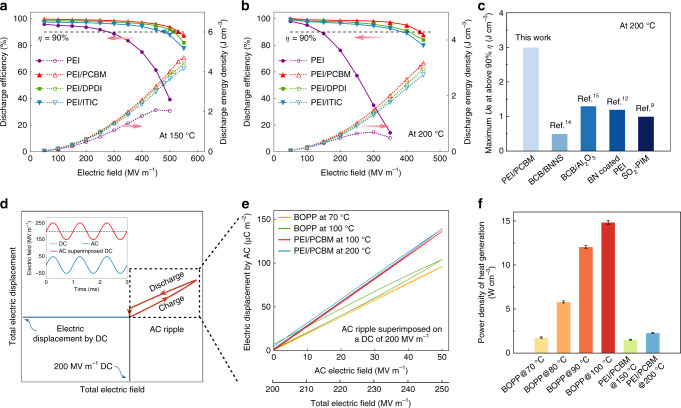
Electric energy storage capability. **a**, **b** Field-dependent energy density and discharge efficiency of PEI and PEI/PCBM (0.5 vol.% PCBM), PEI/DPDI (0.75 vol.% DPDI), and PEI/ITIC (0.25 vol.% ITIC) composites at (**a**) 150 °C and (**b**) 200 °C. **c** Comparison of maximum discharged energy density at above 90% efficiency between the PEI/PCBM and the state-of-the-art polymer-based high-temperature dielectrics at 200 °C. **d** Schematic diagram of the *D*-*E* loop under AC ripple voltage superimposed DC bias. Only the first half AC time period of the loop is shown for simplicity. Inset, the pattern of the applied electric field signal. **e***D*-*E* loops and **f** power density of heat generation for BOPP and PEI/PCBM driven by AC ripple (50 MV m^−1^) superimposed DC bias (200 MV m^−1^) at different temperatures. The average values and max–min error bars of the results were obtained from five parallel samples.

Figure 3 presents the high-temperature energy storage performance derived from the unipolar electric displacement–electric field (*D*-*E*) loops (Supplementary Figs. 18–21). The assessment is focused on the *U*_e_ achieved at a high *η* level (above 90%) to avoid overwhelming device heating by the energy loss in practical applications. Strikingly, the all-organic composites incorporated with different types of molecular semiconductors all exhibit drastically improved high-temperature capacitive performance relative to the pristine PEI. At 150 °C, the *U*_e_ above 90% efficiency of the composites ranges from 3.4 to 4.5 J cm^−3^, whereas that of the pristine PEI is only 1.0 J cm^−3^ (Fig. 3a and Supplementary Figs. 22 and 23). More remarkably, at 200 °C, the maximum *U*_e_ with *η* above 90% of the composites remains as high as 3.0 J cm^−3^ (Fig. 3b), which is 2.3–6.0 times those of the state-of-the-art high-temperature polymer-based dielectrics at the same temperature (0.5–1.3 J cm^−3^) (Fig. 3c). It is noteworthy that this level of energy density above 90% efficiency achieved at 200 °C is even comparable to the room-temperature value (~4.0 J cm^−3^) of BOPP^11^, which implies the all-organic composites are directly applicable in the extreme-environment electrical and electronic systems. In order to testify the generality of this approach, we employed various common heat-resistant dielectric polymers other than the PEI as the matrices, including polyethersulfone (PES), fluorine polyester (FPE) and polyimide (PI). It is evident that the *U*_e_ values with *η* above 90% of the PES, FPE and PI films incorporated with 0.50 vol.% of the PCBM are 3.6, 7.1, and 7.3 times those of the corresponding pristine polymers at 150 °C (Supplementary Fig. 24). The vastly improved energy density at high temperatures could help reduce the capacitor size and enable compact power modules. We also investigated the bipolar loops of the all-organic composite (Supplementary Fig. 25). We found that the energy storage performance derived from the bipolar loops are comparable to those from the unipolar loops (Supplementary Fig. 26), e.g., at 150 °C, the *η* values of PEI/PCBM derived from the unipolar and bipolar loops at 500 MV m^−1^ are 94% and 92%, respectively. These results imply that the materials are suitable for both DC and AC applications.

### Suppression of power dissipation

Another benefit of employing the high-temperature dielectric materials is to ease the thermal management. To exemplify this point, we exerted a constant DC field superimposed with an AC ripple on the materials to simulate the operation of capacitors in the power inverter of hybrid electric vehicles (HEVs) (Fig. 3d), and calculated the heat generation from the energy loss in this process. Note that BOPP capacitors are currently employed in the power inverter to convert the DC power supplied by the battery to the AC power required to drive the traction motor. In the HEVs, an auxiliary cooling system is used to reduce the temperature of the power inverter from ~140 °C to below 70 °C for stable operation of the BOPP capacitors. The *D*-*E* loops of the PEI/PCBM measured under the superimposed DC (200 MV m^−1^) and AC (50 MV m^−1^, 1 kHz frequency) voltage are compared with those of the BOPP films (Fig. 3e). We set the DC electric field at 200 MV m^−1^ in consideration that the capacitor films in power inverter of HEVs are usually around 3 μm in the thickness, upon which a DC voltage of ~600 V is applied. For the AC ripple voltage, it is relatively low in comparison to the DC voltage, and is highly dependent on the working condition of the power control unit. Here we set the AC amplitude at 50 MV m^−1^ for the evaluation. Indeed, BOPP shows a slim loop at 70 °C, characteristic of a low energy loss (i.e., 0.9%). However, at 100 °C the loss reaches 7.1%. Such energy loss is dissipated as Joule heat, and the power density of heat generation can be calculated by *P*_J_ = *U*_l_ × 2*f*_AC_, where *U*_l_ is the energy loss density in half the AC time period and *f*_AC_ is the AC frequency (Fig. 3f). The *P*_J_ of BOPP at 100 °C is 14.8 W cm^−3^, more than eight times that at 70 °C (1.8 W cm^−3^), which can lead to overheating of the device in the absence of active cooling. Interestingly, the PEI/PCBM film has an extremely low loss of 0.5% up to 150 °C. Under this temperature, the *P*_J_ of the PEI/PCBM remains at only 1.5 W cm^−3^, which is even lower than that of BOPP operating at 70 °C. These results suggest that the PEI/PCBM can be directly used in the HEVs without the need of auxiliary cooling systems. Even at 200 °C, the *P*_J_ of the PEI/PCBM estimated from the *D*-*E* loop is still as low as 2.3 W cm^−3^, implying that the material can be used in the HEVs with advanced wide-bandgap semiconductor electronics operating at higher temperatures^5^, e.g., silicon carbide HEVs.

### Film quality

**Fig. 4:**
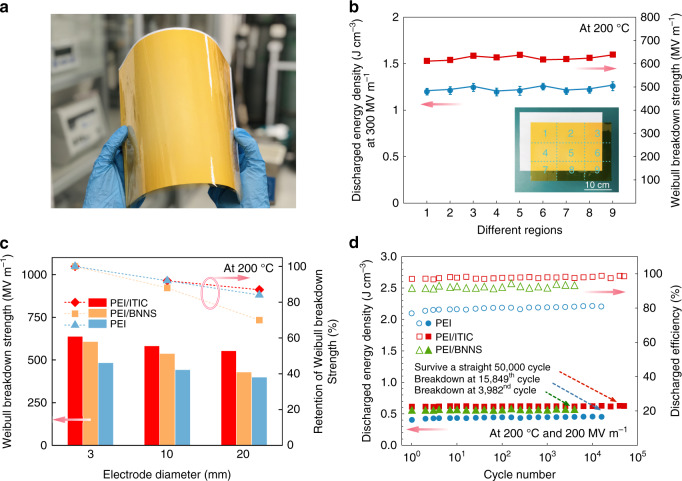
Reliability and stability evaluations. **a** Photograph of a PEI/ITIC composite film (0.25 vol.% ITIC) of 20 × 30 cm^2^ area. An A4 paper sheet is stacked underneath the film as a reference. **b** Characteristic breakdown strength and discharged energy density tested at different regions (as marked in the inset photograph) of the large PEI/ITIC film at 200 °C. The average values and max–min error bars of the results were obtained from five parallel samples. **c** Characteristic breakdown strength and the retention rate with increasing the electrode diameter at 200 °C of PEI, PEI/ITIC (0.25 vol.% ITIC) and PEI/BNNS (10 vol.% BNNS). **d** Cyclic performance of PEI, PEI/ITIC (0.25 vol.% ITIC) and PEI/BNNS (10 vol.% BNNS) measured with 20-mm-diameter electrode at 200 °C and 200 MV m^−1^ electric field.

Given the fact that dielectric polymers for electrostatic capacitors are in the form of continuous films with very few defects, we finally evaluated the ability of the all-organic composites to form uniform and high-quality films. We cast a large all-organic composite film of 20 × 30 cm^2^ area from PEI/ITIC (Fig. 4a), and measured the breakdown strength and discharged energy density at different film regions at 200 °C. The results show a high consistency across the different film regions (Fig. 4b), suggesting that the film is overall uniform. We also analyzed the performance with increased electrode diameters to see if the molecular semiconductors deteriorated the film quality (Fig. 4c, Supplementary Figs. 27 and 28 and Supplementary Note 6). Clearly, the retention of the characteristic breakdown strength of the PEI/ITIC film as the testing electrode diameter increases from 3 to 20 mm is 87%, which is comparable to that of the pristine polymer film (83% retention). This result implies that the molecular semiconductors have no perceptible adverse impact on the film quality. By contrast, the PEI/BNNS nanocomposite shows only 70% retention of the breakdown strength. This is likely due to the presence of particle agglomeration and imperfect polymer/particle interface associated with the large concentration of inorganic nanofillers. The all-organic composite also outperforms the pristine polymer and the PEI/BNNS in the cyclic charge–discharge tests (Fig. 4d), indicative of greater long-term reliability. These experimental results indicate that the dielectric polymers and the molecular semiconductors are well miscible with each other, and serve to emphasize the advantage of the all-organic composites in forming large-area and high-quality films, which represents a substantial advance over the conventional polymer nanocomposites containing inorganic inclusions.

## Discussion

Two competing factors associated with the molecular semiconductors are speculated to be relevant to the electrical resistivity of the all-organic composite materials, i.e., (1) The trapping of free charges by the molecular semiconductors, and (2) The electronic conduction of the charges excited from the valence band to the conduction band of the molecular semiconductors. The charge trapping is a result of the high-electron affinity of the molecular semiconductors, while the electronic conduction of excited charges is determined by the energy bandgap of the molecular semiconductors. At very low volume concentrations, the high-electron-affinity molecular semiconductors can trap most of the free electrons in the composites, and the electrical resistivity increases with increasing the concentration of the molecular semiconductors due to the increased population density of trapping sites. With further increasing the concentration of the molecular semiconductors, the mean spacing between the adjacent molecular semiconductors becomes smaller and smaller, and the probability of electronic conduction of the excited charge carriers becomes significant, which begins to dominate the electrical property. Therefore, the optimum performance seen at particular concentrations of the molecular semiconductors (Supplementary Figs. 6 and 22) could be a result of the competition of the aforementioned two factors. The reason to the moving peak volume concentration for different molecular semiconductors is most likely due to their distinct energy bandgap values, i.e., 1.7, 2.3, and 2.5 eV for ITIC, PCBM, and DPDI, respectively. A larger bandgap of the molecular semiconductor would lead to shift of the peak towards higher volume concentration because the charge carriers need a higher energy to be excited from the valence band to the conduction band and thus the electronic conduction is of a lower probability to occur at the same volume concentration.

In conclusion, this study offers a novel material strategy for tailoring the high-temperature capacitive performance of dielectric polymers. Further improvement of temperature capability and performance can be accomplishable through tuning the combinations between the molecular semiconductors and dielectric polymers other than those reported in this study. In view of the numerous existing high-electron-affinity compounds, this approach is highly applicable. Since the raw materials are readily accessible, and the film processing is straightforward and simple, this all-organic composite strategy shall be able to address the challenge in scalable fabrication of high-performance, large-area and high-quality polymer films required for high-temperature dielectric energy storage.

## Methods

### Materials

Unless otherwise noted, commercially available reagents were used without further purification. The BOPP and the heat-resistant polymer dielectrics polyetherimide (PEI, *T*_g_ ≈ 217 °C), fluorene polyester (FPE, *T*_g_ ≈ 320 °C) and polyethersulfone (PES, *T*_g_ ≈ 218 °C) were provided by PolyK technologies. The BOPP is capacitor grade film with the thickness of 4.8 μm. The polyimide (PI, *T*_g_ ≈ 360 °C) was synthesized from the raw materials pyromellitic dianhydride (PMDA) and 4,4′-oxydianiline (ODA), both of which were purchased from Sigma-Aldrich.

The three kinds of molecular semiconductors ITIC (2,2′-[[6,6,12,12-Tetrakis(4-hexylphenyl)-6,12-dihydrodithieno[2,3-d:2′,3′-d′]-s-indaceno[1,2-b:5,6-b′]dithiophene-2,8-diyl]bis[methylidyne(3-oxo-1H-indene-2,1(3H)-diylidene)]]bis[propanedinitrile]), PCBM ([6,6]-Phenyl C61 butyric acid methyl ester), DPDI (2,2′,9,9′-Tetrakis(1-pentylhexyl)-[5,5′-bianthra[2,1,9-def:6,5,10-d′e′f′]diisoquinoline]-1,1′,3,3′,8,8′,10,10′(2H,2′H,9H,9′H)-octone) were all purchased from Sigma-Aldrich.

The chemical structures of the dielectric polymers and the molecular semiconductors are shown in Supplementary Fig. 29.

### Preparation

The all-organic composite films were prepared by a solution casting method. The molecular semiconductor powders (ITIC, PCBM or DPDI) of a proper weight according to the desired concentration in the composites were first dissolved in 10 mL N-Methyl pyrrolidone (NMP). Then 400 mg of the heat-resistant polymer pellets/powders (PEI, FPE or PES) were dissolved in 10 mL of NMP and stirred for 2 h. Afterwards the NMP solution of the molecular semiconductors was mixed with the polymer solution, and the mixture was first stirred for 5 min and then sonicated for 60 min using a tip-type sonicator (150 W). The solution was then drop cast onto a clean glass slide. The cast films were kept in a drying oven at 80 °C for 12 h to remove the solvent and then heated to 125 °C and 150 °C for 1 h, respectively, followed by a final drying step at 200 °C for 12 h. Afterwards, the films were kept in a vacuum oven at 200 °C for one day to further remove any residual solvent. Finally, the films were peeled off from the glass substrate after soaking in deionized water for 30 s, and dried in a vacuum oven at 100 °C for 12 h. The typical thickness of the films used for electrical characterizations is within the range of 10–12 μm.

The pure PI films were prepared following a literature method^31^. The PI/PCBM composite films were prepared in a process described as follows. First, a poly(amic acid) (PAA) solution was prepared by dissolving the ODA monomer in N,N-dimethyl-acetamide (DMAc). An equimolar amount of PMDA was then added stepwise to the ODA-DMAc solution to ensure complete dissolution, and was stirred for 12 h to yield a PAA solution. Afterwards the PCBM powders were dispersed in DMAc and sonicated for 120 min using a tip-type sonicator (100 W). The PAA solution was then added into the suspension and sonicated for 12 h to yield a PAA/PCBM suspension. The suspension was then cast onto a glass slide and dried at 80 °C for 4 h. After that, the composite was thermally treated in a muffle furnace by the gradient heating method, from room temperature to 100, 130, 160, 190, 230, 260, 290, 320, and 350 °C for half an hour. Subsequently, the films were cooled to room temperature, and placed in a vacuum oven at 200 °C for 12 h to remove any residual solvent before being peeled off from the glass slide and dried in a vacuum oven at 80 °C for 4 h.

BNNSs were prepared from the hexagonal boron nitride powders (Sigma-Aldrich) using a solution phase exfoliation method^32^. The PEI/BNNS nanocomposite films were prepared in a process similar to that in the preparation of the all-organic composites except for replacing the molecular semiconductors with the BNNSs.

The samples for the transmission electron microscope (TEM) study were prepared in a process described as follows. For the PCBM sample, NMP solution of the PCBM (0.02 mg mL^−1^) was first stirred for 5 min and then sonicated for 60 min using a tip-type sonicator (150 W). Afterwards, a few drops of the solution were placed on an ultra-thin carbon film deposited on one side of the copper grid, and the samples were kept in a vacuum oven at 100 °C for 12 h before TEM observation. For the PEI/PCBM sample, the process is similar to that of the PCBM except that NMP solution of the PEI (1 mg mL^−1^) blended with a proper concentration of the PCBM was used as the precursor (0.5–2 vol.% to the PEI).

The samples for the KPFM measurement were prepared in a process described as follows. First, the NMP solution of PEI mixed with PCBM was used to cast a film on a customized substrate with a pair of electrodes (Supplementary Fig. 12) by spin-coating. The thickness of the coated PEI/PCBM film can be controlled by changing the solution concentration and the experimental parameters of spin-coating. Typically, the NMP solution of PEI (1 mg mL^−1^) mixed with PCBM (0.005–0.01 mg mL^−1^) spin-coated at 500–700 r.p.m. yields a film of 50–100 nm thickness on a heated spin platform (80 °C). The redundant PEI/PCBM on the electrodes surface was washed off by the NMP, and the PEI/PCBM between the electrodes was dried in a vacuum oven at 200 °C for 12 h. The customized substrate was made from Si wafer with 2 × 1 cm^2^ in size, and a dense SiO_2_ insulation layer with a thickness of 2 µm covered on the Si substrate. The electrodes were fabricated on the SiO_2_ insulation layer by vacuum deposition followed by photolithography with 5 × 5 mm^2^ in size, and the gap between the two electrodes is 1 mm (Supplementary Fig. 12). The aluminum metal was selected as the electrode material because of its suitability for this fabrication process. According to the size of the substrate and the test requirements, a printed circuit board (PCB) was designed for circuit connections on the substrate. The substrate was fixed in the middle of the PCB and the electrodes were connected to the external circuit by conductive silver adhesive. A DC voltage can be applied on the electrodes by the external voltage source, which can generate a relatively uniform electric field between the electrodes.

### Characterization

FTIR spectra were measured in the attenuated total reflectance (ATR) mode with a Thermo Scientific Nicolet iS10 spectrometer. Dynamic mechanical analysis (DMA) was performed by using a TA Instruments DMAQ800 at a heating rate of 5 °C min^−1^. Atomic force microscopy (AFM) images of the surface morphology were taken with a Bruker Dimension Icon atomic force microscope in tapping mode. For the KPFM measurement and the PEA measurement, see Supplementary Notes 1 and 2 and Supplementary Fig. 15 for details. TEM images were obtained on a JEOL JEM-2010F. Extra high-magnification spherical aberration corrected TEM measurements were performed with a FEI Titan 80–300. Dielectric spectra were conducted over a broad frequency (10^2^–10^6^ Hz) using a Novocontrol Concept 80 dielectric spectroscopy meter in conjunction with a Quatro-Cryosystem temperature control system. Conduction currents were measured under various electric fields provided by a Keithley 6517B pA meter/voltage source. TSDC was measured using Keithley 6517B electrometer with the following recipe. The samples were first polarized under 250 MV m^−1^ DC electric field at 230 °C for 1 h, and then were rapidly cooled down to −10 °C with the applied electric field. Afterwards, the samples were placed at −10 °C for 5 min and then the electric field was removed. Finally, the samples were short circuited and heated to 250 °C at the heating rate of 2 °C min^−1^ with the current recorded. Ultraviolet photoelectron spectroscopy (UPS) was carried out on a Thermo Scientific ESCALab 250Xi with the He-I lamp radiation (21.2 eV) and the Fermi level was calibrated using clean Au. UV–vis spectroscopy was obtained on a Hitachi U-3010 spectrophotometer to determine the UV–vis absorption spectra of the samples. Optical transmittance of the samples in the wavelength range 200–700 nm was measured with wavelength accuracy of ±0.3 nm. Electric displacement–electric field loop (*D*-*E* loop) tests were performed by using K-CPE1901-AI-30kV high-voltage test system with artificial intelligence control (PolyK Technologies). Charged and discharged energy densities were calculated from the *D*-*E* loops using a way illustrated in Supplementary Fig. 18. The samples were soaked in dimethylsilicone fluid and subjected to a triangular unipolar wave with a frequency of 10 Hz. The test control system is PK-CPE1901 and the high-voltage amplifier is Trek PA05035. The maximum output voltage is ±30 kV, the bandwidth is 30 kHz, the slew rate is 550 V µs^−1^, and the voltage gain is 3000:1. Ripple measurements with DC bias for electrical vehicles were performed by the PK-CPE1901 test system (PolyK Technologies) to simulate the operation of DC-link capacitors used in the power inverter of HEVs. The specimens were subjected to a sine wave bipolar voltage with a frequency of 1000 Hz on top of a high DC bias, and the *D*-*E* loops were recorded. The cyclic charge–discharge tests were performed using a PK-CPE1901 test system (PolyK Technologies). Trek PA05035 high-voltage amplifier was used to charge the capacitor sample soaked in dimethylsilicone fluid and the typical charging time was ∼100 ms. Dielectric breakdown strength measurements were performed on a TREK 610 C amplifier, where a constant DC voltage ramp of 500 V s^−1^ was applied to the test sample until the electrical failure, and the experimental data were analyzed using the two-parameter Weibull statistic (Supplementary Information). Gold electrodes of a diameter of 3 mm (unless otherwise noted) and a thickness of 60 nm were sputtered on both sides of the polymer films for the measurements of dielectric spectrum, conduction current, *D*-*E* loop and breakdown strength.

### Electrostatic potential simulation

The spatial distribution of electrostatic potential for molecular semiconductor models is determined by DFT. DFT is based on the first-principle calculation and the wave functions of calculated molecules are determined according to the solution of basic Schrodinger’s equation. In this work, DFT calculations with the B3LYP hybrid function and the 6–31 G (d) basis function are implemented in the Gaussian 09. In the quantitative analyses of electrostatic potential on van der Waals surface in Multiwfn program 3.7, the grid spacings were set to 0.25 bohr^33^. The van der Waals surface referred in this paper denotes the isosurface of electron density 0.001 e/bohr^3^. The color mapped isosurface of electrostatic potential distribution and area percentage in each electrostatic potential range can be obtained.

## Supplementary information

Supplementary Information

## Data Availability

The data that support the findings of this study are available from the authors on reasonable request.
