# Supplementary material for: Polymer/molecular semiconductor all-organic composites for high-temperature dielectric energy storage
Source: Nat Commun. 2020 Aug 6;11:3919. doi: 10.1038/s41467-020-17760-x (PMC7411043; doi:10.1038/s41467-020-17760-x)
Supplement: Supplementary file 1 — Supplementary Information [file 41467_2020_17760_MOESM1_ESM.pdf]

Supplementary Information

Polymer/molecular semiconductor all-organic composites for high-temperature dielectric energy storage

Yuan et al.

Supplementary Figures

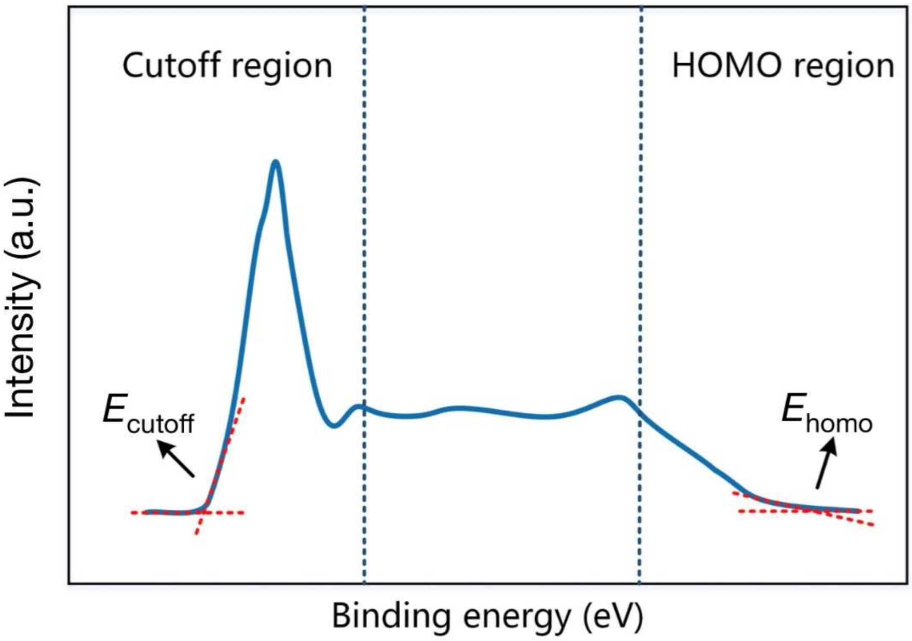

Supplementary Figure 1. Schematic illustration of the ultraviolet photoelectron spectrum for a dielectric polymer. In the UPS measurement, the electrons in the occupied state are excited by the incident photo energy ($h\nu$ = 21.2 eV). The work function (ϕ_d) is calculated by the difference between the incident photoelectron energy and the energy of secondary electron cutoff (E_{cutoff})[1]. The position of E_{cutoff} can be extracted from the intersection of the baseline and the tangents of the curve in the secondary electron cutoff region. E_{homo} is the minimum binding energy of photoelectron from the dielectric (HOMO stands for the highest occupied molecular orbital of the dielectric). The position of E_{homo} can be extracted from the intersection of the baseline and the tangents of the curve in HOMO region[2, 3]. The ionization potential (IP) can be obtained by the summation of ϕ_d and E_{homo}.

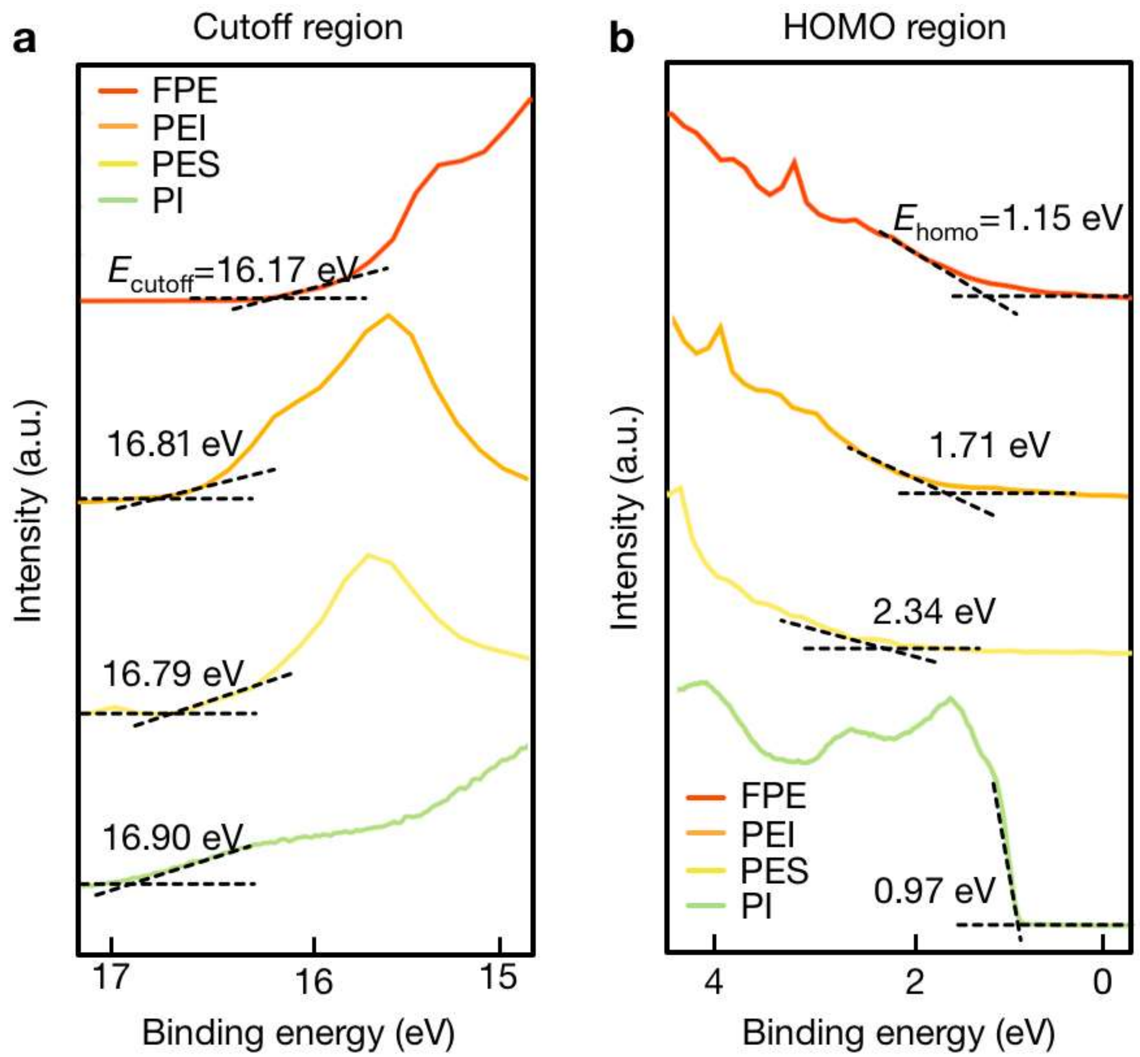

Supplementary Figure 2. UPS measurement of the secondary electron cutoff region (a) and HOMO region (b) of the FPE, PEI, PES and PI polymers. The E_{cutoff} values of FPE, PEI, PES and PI are 16.17 eV, 16.81 eV, 16.79 eV and 16.90 eV, respectively. The work function (ϕ_d), calculated by the difference between the incident photoelectron energy (21.2 eV) and E_{cutoff}, are 5.03 eV, 4.39 eV, 4.41 eV and 4.3 eV for FPE, PEI, PES and PI, respectively. The E_{homo} values of FPE, PEI, PES and PI are 1.15 eV, 1.71 eV, 2.34 eV and 0.97 eV, respectively. The IP values (ϕ_{IP}), calculated by the summation of ϕ_d and E_{homo}, are 6.18 eV, 6.10 eV, 6.75 eV and 5.27 for FPE, PEI, PES and PI, respectively.

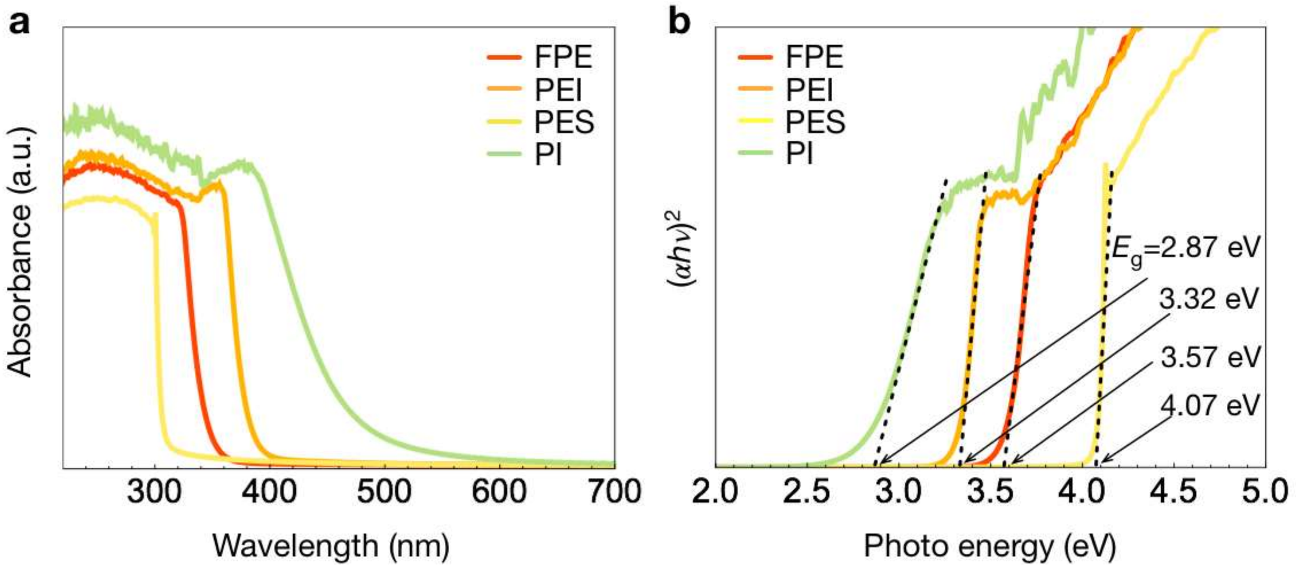

Supplementary Figure 3. UV-vis absorption spectra (a) and $(\alpha h\nu)^2$ - $h\nu$ plots (b) of FPE, PEI, PES and PI dielectric polymers. The curve of $(\alpha h\nu)^2$ versus $h\nu$ is converted from the UV-vis spectrum by Tauc plot, in which α, h, and ν are the absorption coefficient, Planck constant, and light frequency, respectively[1]. The band gap (ϕ_g) determined through the absorption onset of the linear region, are 3.57 eV, 3.32 eV, 4.07 eV and 2.87 eV for FPE, PEI, PES and PI, respectively.

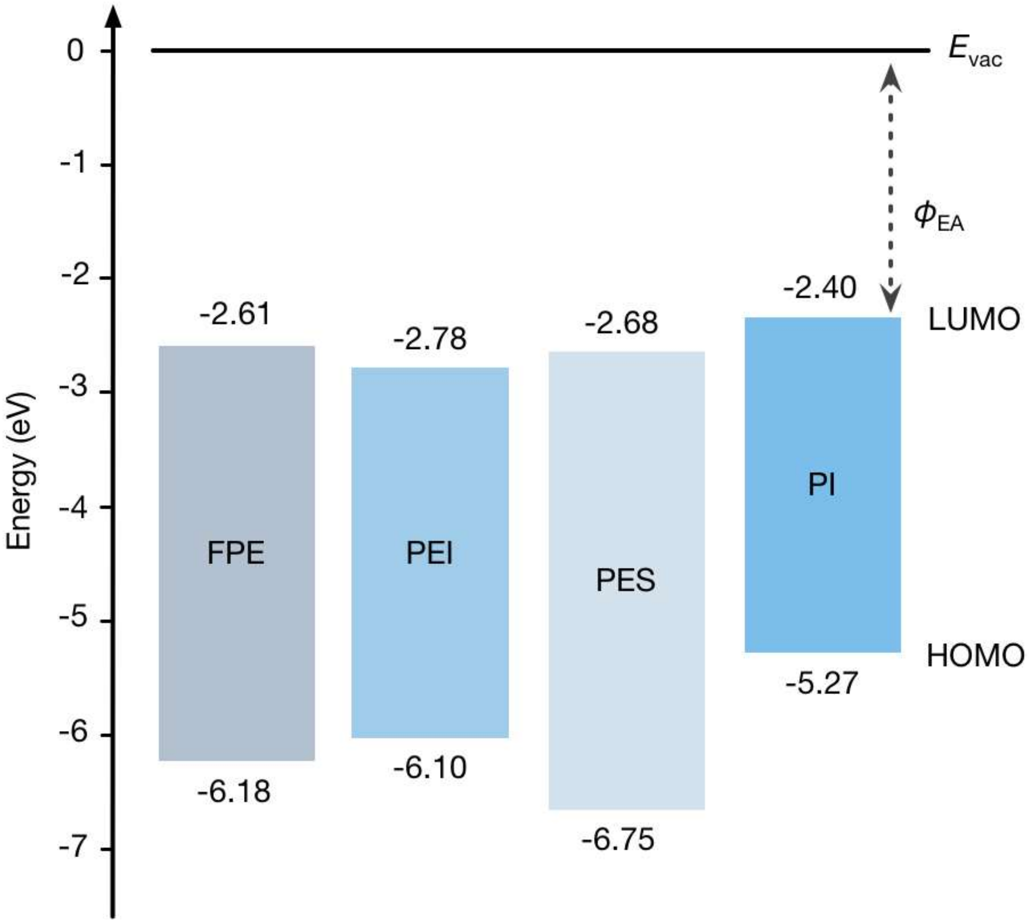

Supplementary Figure 4. Energy band diagram for FPE, PEI, PES and PI dielectric polymers. ϕ_{EA}, electron affinity; E_{vac}, vacuum level. The ionization potential (ϕ_{IP}) is obtained by the UPS measurement in Supplementary Figure 2. With the obtained ϕ_{IP}, the position of the HOMO level can be determined. The band gap (ϕ_g) is obtained by the UV-vis measurement in Supplementary Figure 3. Thus, the position of LUMO (lowest unoccupied molecular orbital) levels, calculated by the difference between the HOMO level and the band gap, are 2.61 eV, 2.78 eV, 2.68 eV and 2.40 eV for FPE, PEI, PES and PI, respectively, which are also their respective electron affinity (ϕ_{EA}). Energy values are reported as absolute values relative to a vacuum.

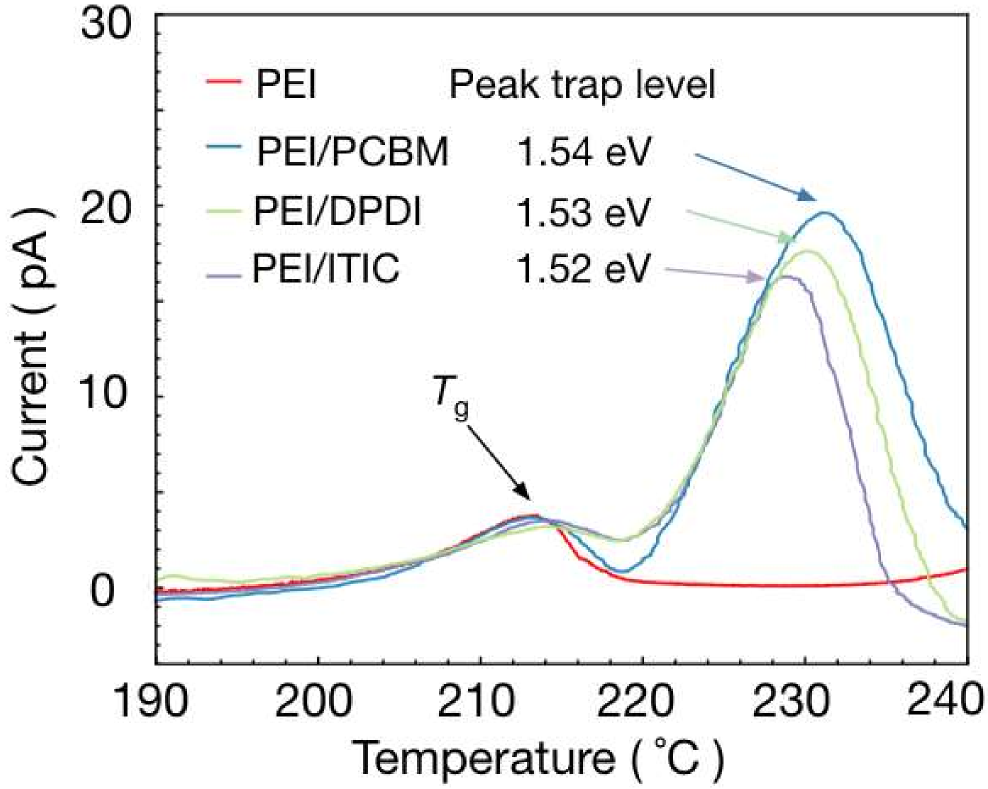

Supplementary Figure 5. TSDC curves of PEI, PEI/ITIC, PEI/PCBM and PEI/DPDI. The trap energy levels are determined using a literature approach[4].

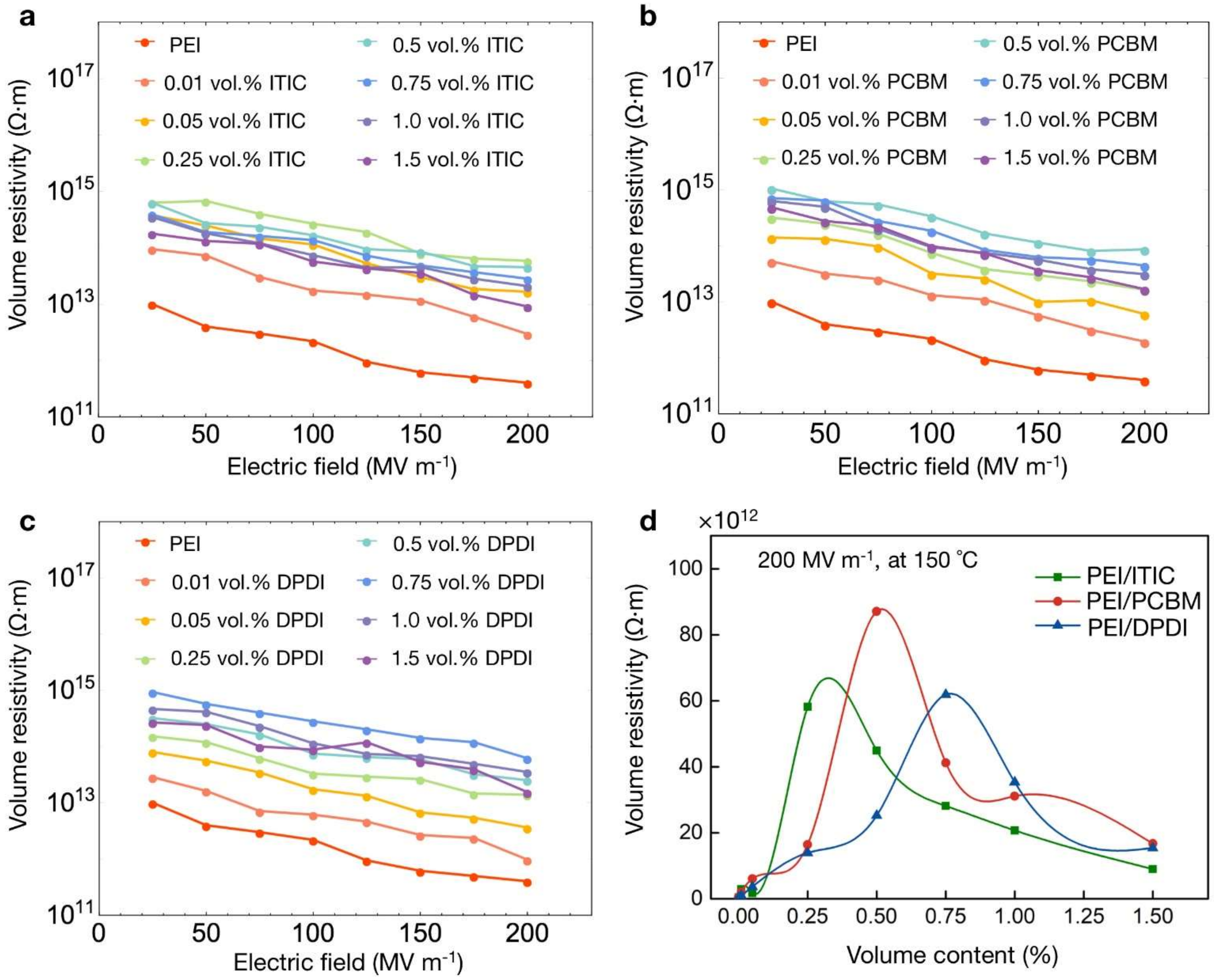

Supplementary Figure 6. Volume resistivity of the PEI-based all-organic composites with varied contents of (a) ITIC, (b) PCBM and (c) DPDI as a function of the applied electric field at 150 °C. (d) Summary of the volume resistivity of the PEI-based all-organic composites as a function of the concentration of the molecular semiconductors at 200 MV m^{-1} and 150 °C.

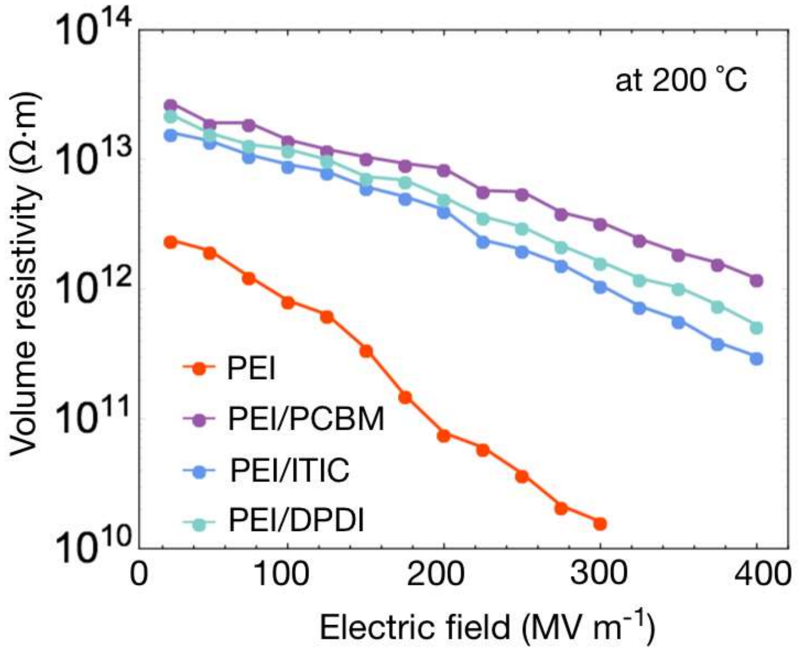

Supplementary Figure 7. Volume resistivity of the PEI/PCBM (0.5 vol.% PCBM), PEI/DPDI (0.75 vol.% DPDI), and PEI/ITIC (0.25 vol.% ITIC) composites as a function of the applied electric field at 200 °C.

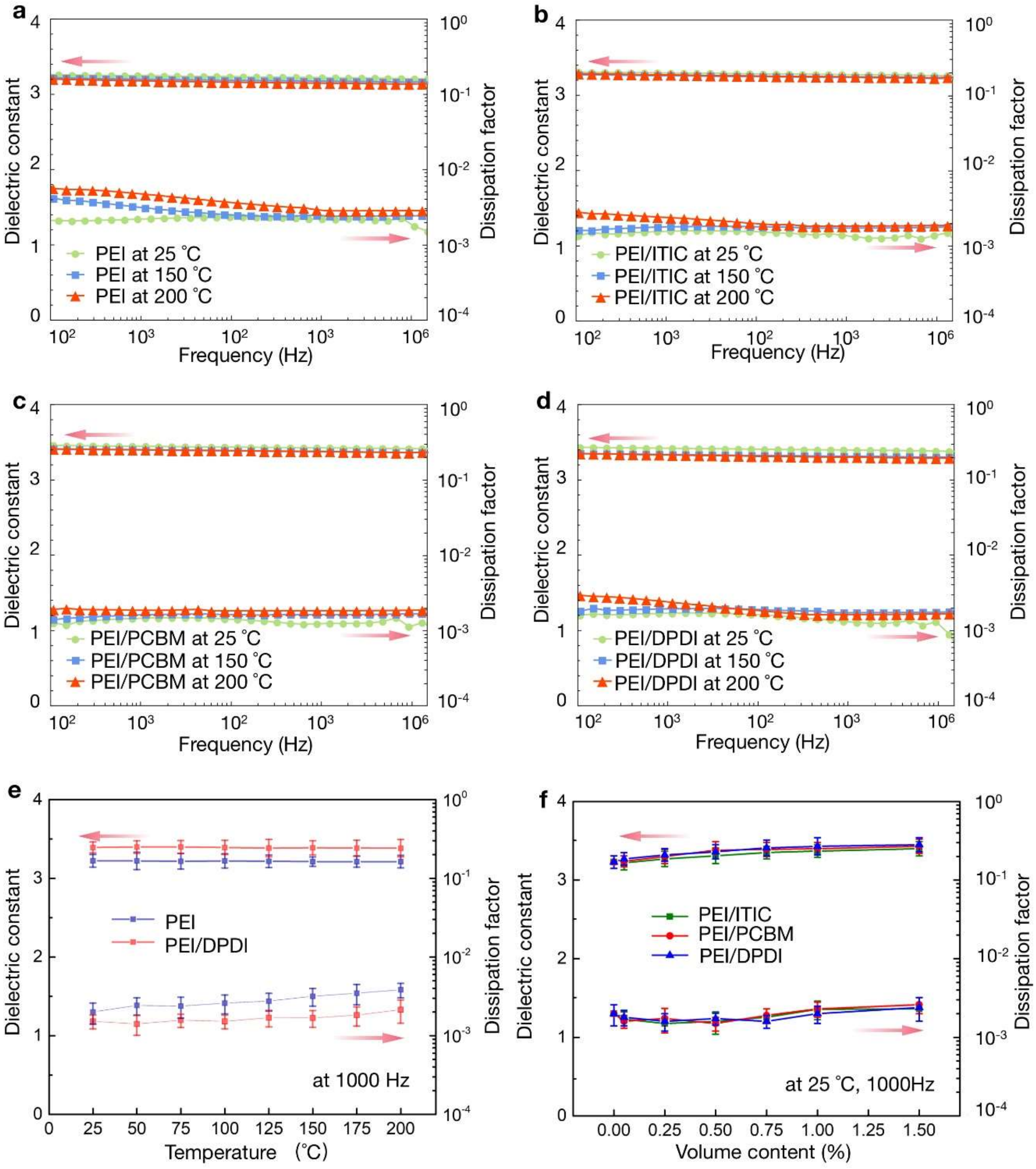

Supplementary Figure 8. Frequency dependence of the dielectric constant and dissipation factor of (a) PEI, (b) PEI/ITIC (0.25 vol.% ITIC), (c) PEI/PCBM (0.5 vol.% PCBM) and (d) PEI/DPDI (0.75 vol.% DPDI) at different temperatures. (e) Temperature-dependent dielectric constant and dissipation factor of PEI and the all-organic composite. (f) Dielectric constant and dissipation factor of the all-organic composites as a function of the ITIC, PCBM and DPDI content. The average values and max–min error bars of the results were obtained from five parallel samples.

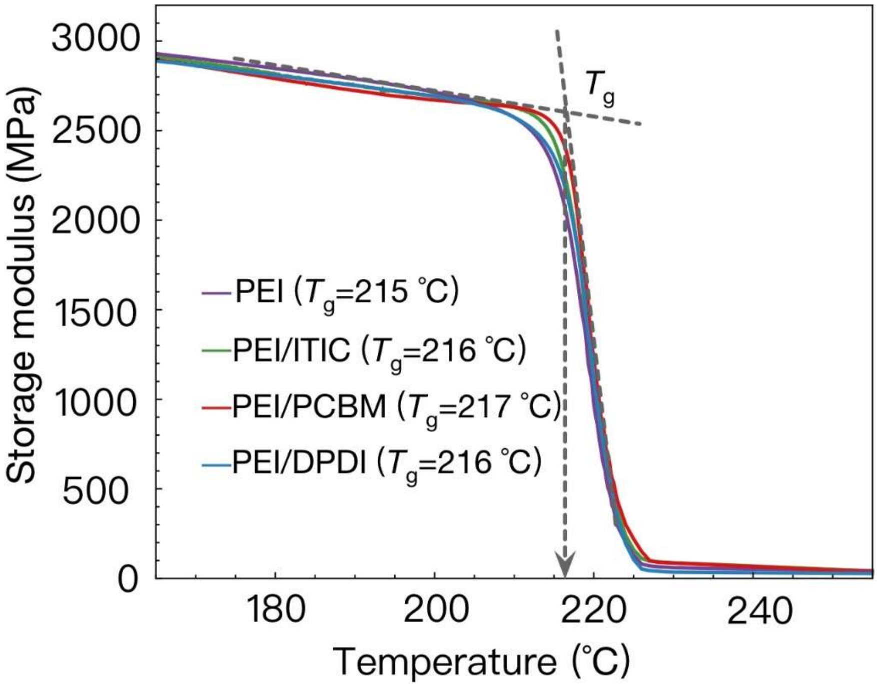

Supplementary Figure 9. DMA curves of PEI, PEI/ITIC, PEI/PCBM and PEI/DPDI at 1 Hz frequency. The storage modulus was recorded from 155 to 250 °C with a heating rate of 5 °C min^{-1}. Glass transition temperatures T_g for PEI, PEI/ITIC, PEI/PCBM and PEI/DPDI were measured from the storage modulus profiles.

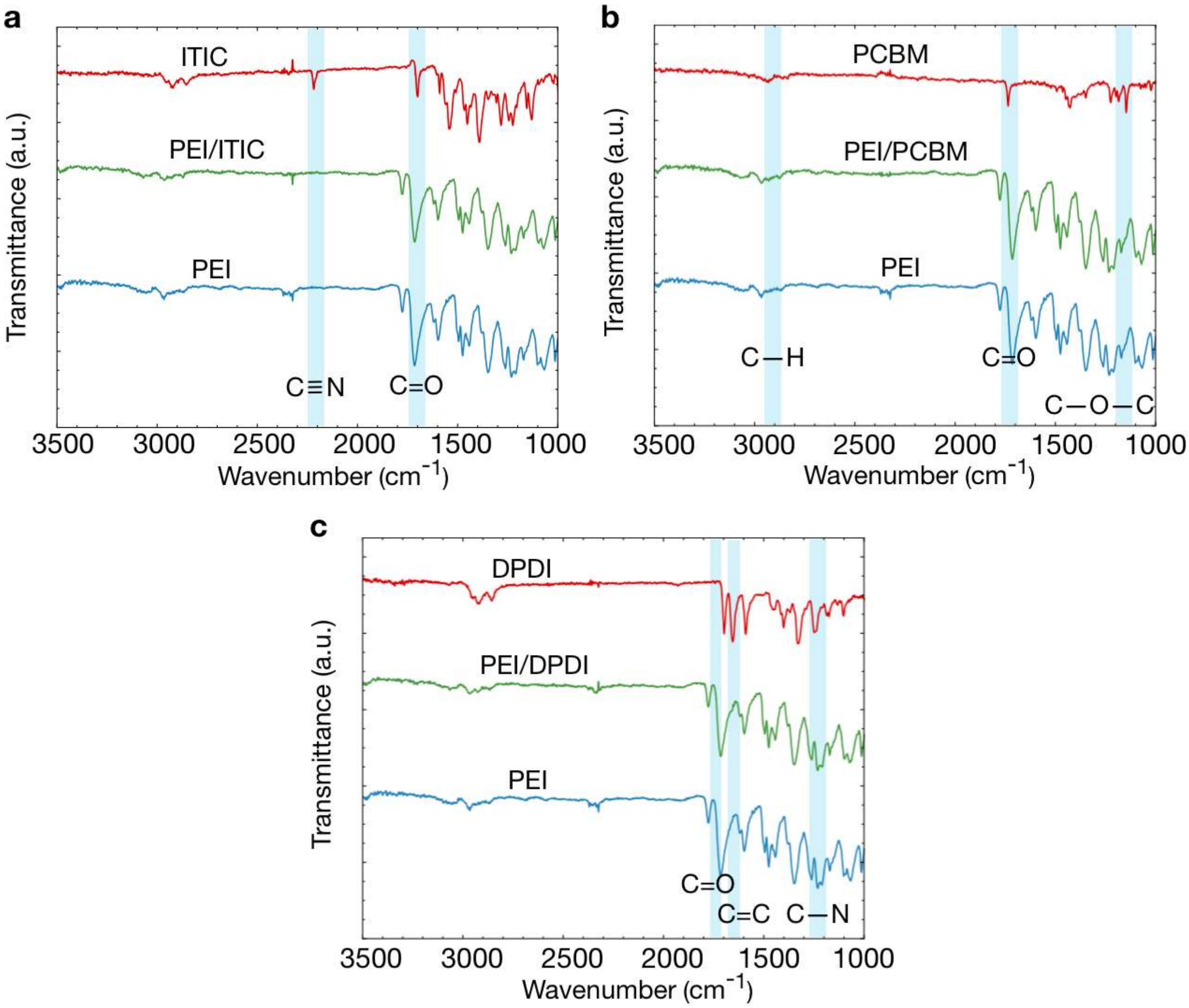

Supplementary Figure 10. ATR-FTIR spectra of (a) ITIC, (b) PCBM, (c) DPDI and the PEI-based composites. For ITIC, the characteristic peaks shown in the spectra include the cyanide group (C≡N) at 2252 cm^{-1} and the carbonyl group (C=O) at 1740 cm^{-1}. For PCBM, the characteristic peaks shown in the spectra include the (C−H) stretching at 2940 cm^{-1}, the carbonyl group (C=O) at 1740 cm^{-1} and the ether group (C−O−C) at 1144 cm^{-1}. For DPDI, the characteristic peaks shown in the spectra include the carbonyl group (C=O) at 1740 cm^{-1}, the carbon-carbon double bond group (C=C) at 1620 cm^{-1} and the carbon-nitrogen bond group (C−N) at 1144 cm^{-1}. These characteristic peaks of molecular semiconductor are almost invisible in the PEI/ITIC, PEI/PCBM and PEI/DPDI composite dielectrics, which can be attributed to the ultra-low concentrations of the molecular semiconductors and the fact that their main characteristic peaks overlap with the characteristic peaks of PEI. The characteristic peaks in PEI for the imide (carbonyl

and amine stretching in the phthalimide rings), ether (aryl–ether–aryl stretching) and propylidene groups (carbon–hydrogen stretching in the aromatic rings) were successfully identified. It is clear to see that the FTIR spectra of PEI, PEI/ITIC, PEI/PCBM and PEI/DPDI are almost identical, which suggests that the chemical structure of the host dielectric polymer was unaffected by the incorporated molecular semiconductors.

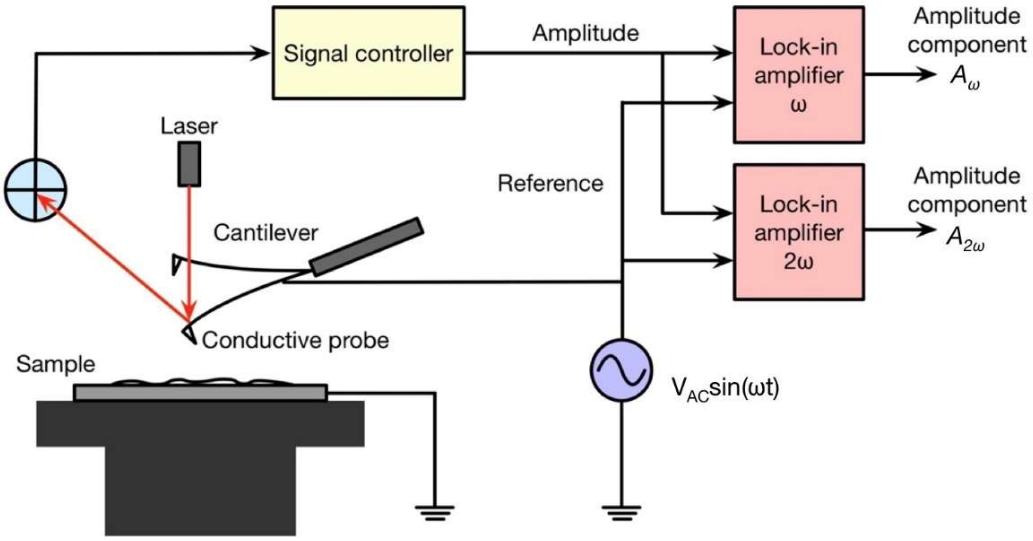

Supplementary Figure 11. The schematic diagram of the working principle of the PeakForce KPFM for local surface potential measurement.

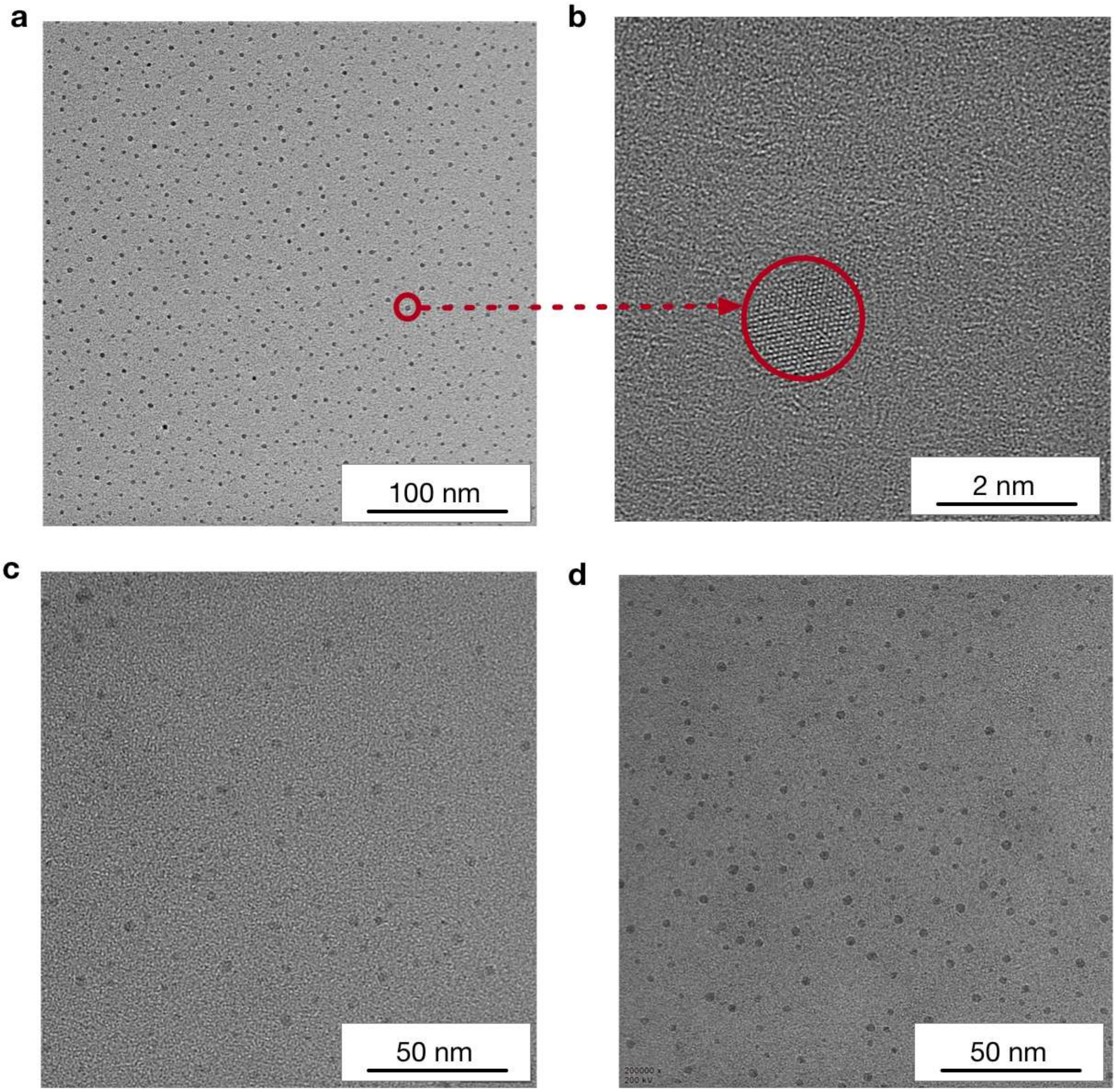

Supplementary Figure 12. (a) Low-magnification TEM image and (b) high-magnification spherical aberration corrected TEM image for the PCBM clusters. The PEI/PCBM composite films cast on the ultra-thin carbon film deposited on one side of the copper grid with (c) low concentration (0.5 vol.%) and (d) high concentration (2 vol.%) of the embedded PCBM clusters. The PCBMs that tend to assemble into clusters of about 2 nm in diameter are found to be uniformly dispersed in the polymer matrix at either the low or the high concentrations.

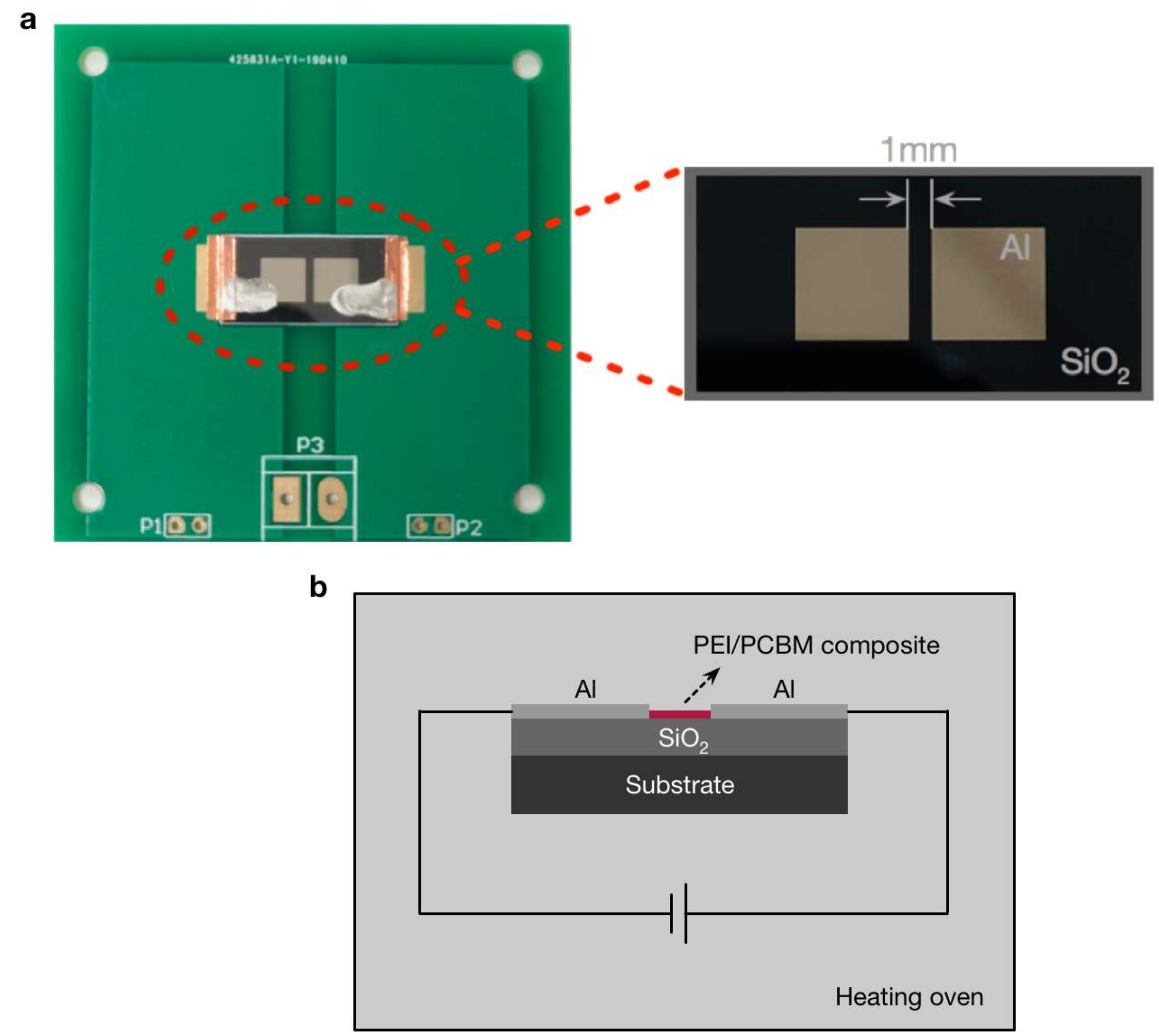

Supplementary Figure 13. (a) Digital photographs of the PCB with aluminum electrodes on a customized substrate for circuit connections in the KPFM measurement. (b) The schematic diagram showing the external circuit for implementing the charge injection prior to the KPFM measurement.

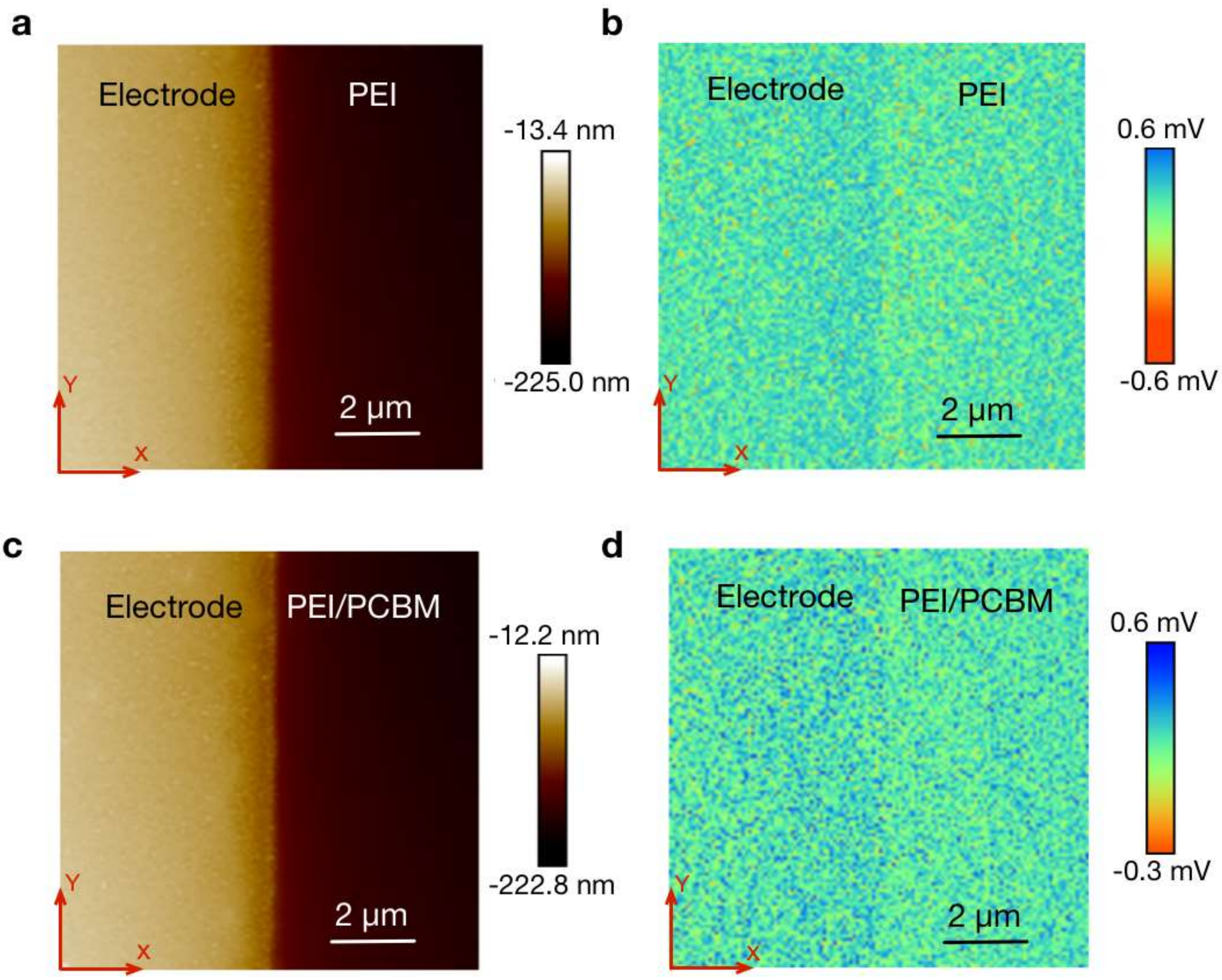

Supplementary Figure 14. (a) Topography and (b) surface potential of the pristine PEI. (c) Topography and (d) surface potential of the PEI/PCBM composite, and no charge injection was implemented prior to the measurement.

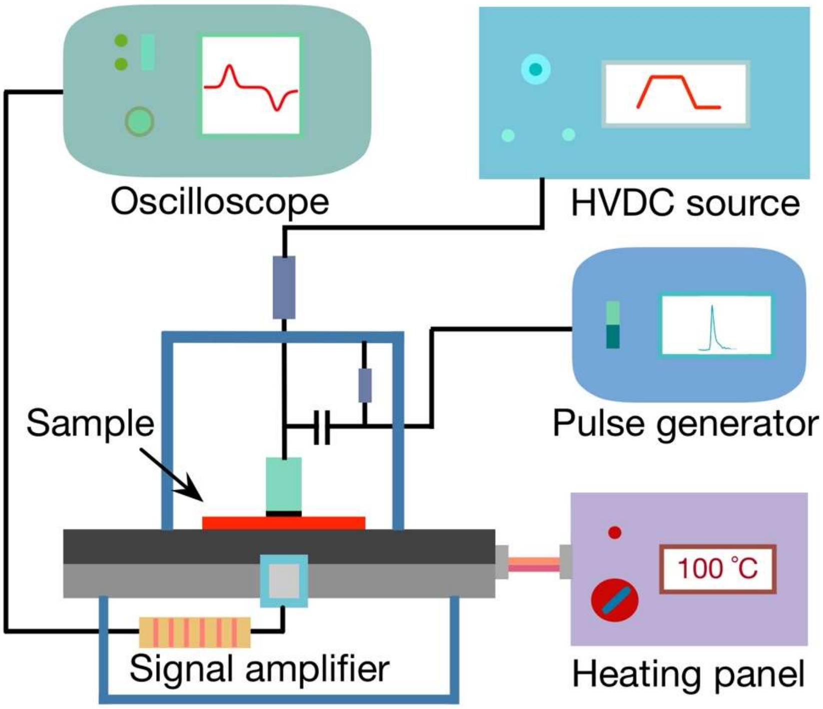

Supplementary Figure 15. Schematic representation of the home-made PEA test system. The space charge behaviour of film samples was studied through the pulse electro-acoustic (PEA) method[9] with a home-made test system that has the high-temperature operating ability up to 100 °C. Film samples with the thickness of about 100 μm were used. The electrodes were made of aluminum, and the top electrode was covered by a semi-conducting film to improve the acoustic match. A poly(vinylidene fluoride) (PVDF) film with the thickness of 5 μm was used as the piezoelectric sensor to obtain an optimized signal strength. The amplifier, produced by MITEQ, had the bandwidth of 0.01~500 MHz and maximum amplification of 47 dB. The applied excitation voltage pulse was 1 kV with 5 ns width. As compared to the applied DC stresses of 200 MV m^{-1}, the electric field caused by the testing pulses was much smaller, therefore, the impact of the testing pulse was limited. Before measurements, the samples were heated at 100 °C in the vacuum oven for 1 hour while electrically short-circuited to eliminate the charges generated during processing. The tests were performed under voltage-on and voltage-off conditions. The voltage-on test was performed for 8 minutes, thereafter, the voltage-off test continued for 2 minutes. And the results are shown in Supplementary Figure 16.

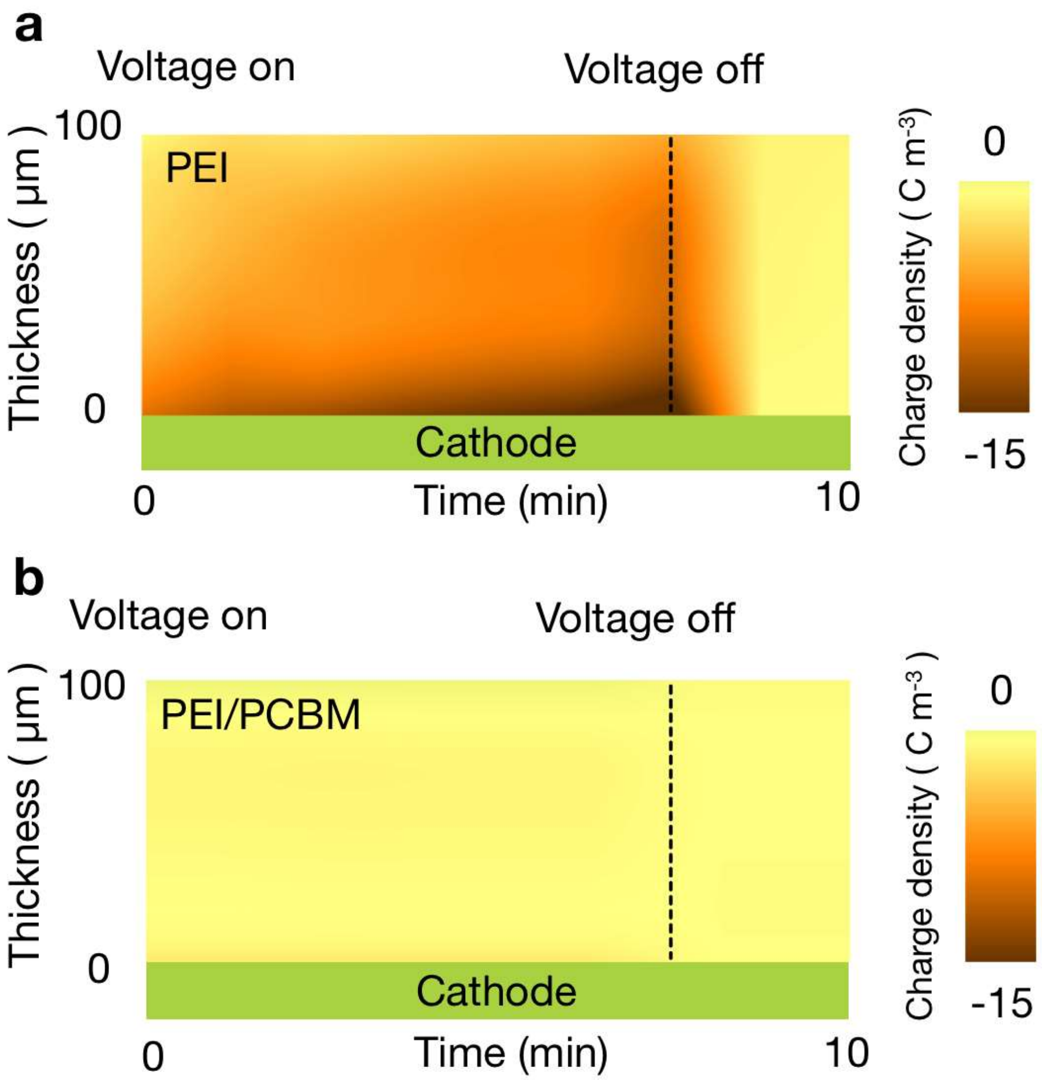

Supplementary Figure 16. Charge injection dynamics of (a) PEI and (b) PEI/PCBM measured by the PEA method under 200 MV m^{-1} DC electric field at 100 °C.

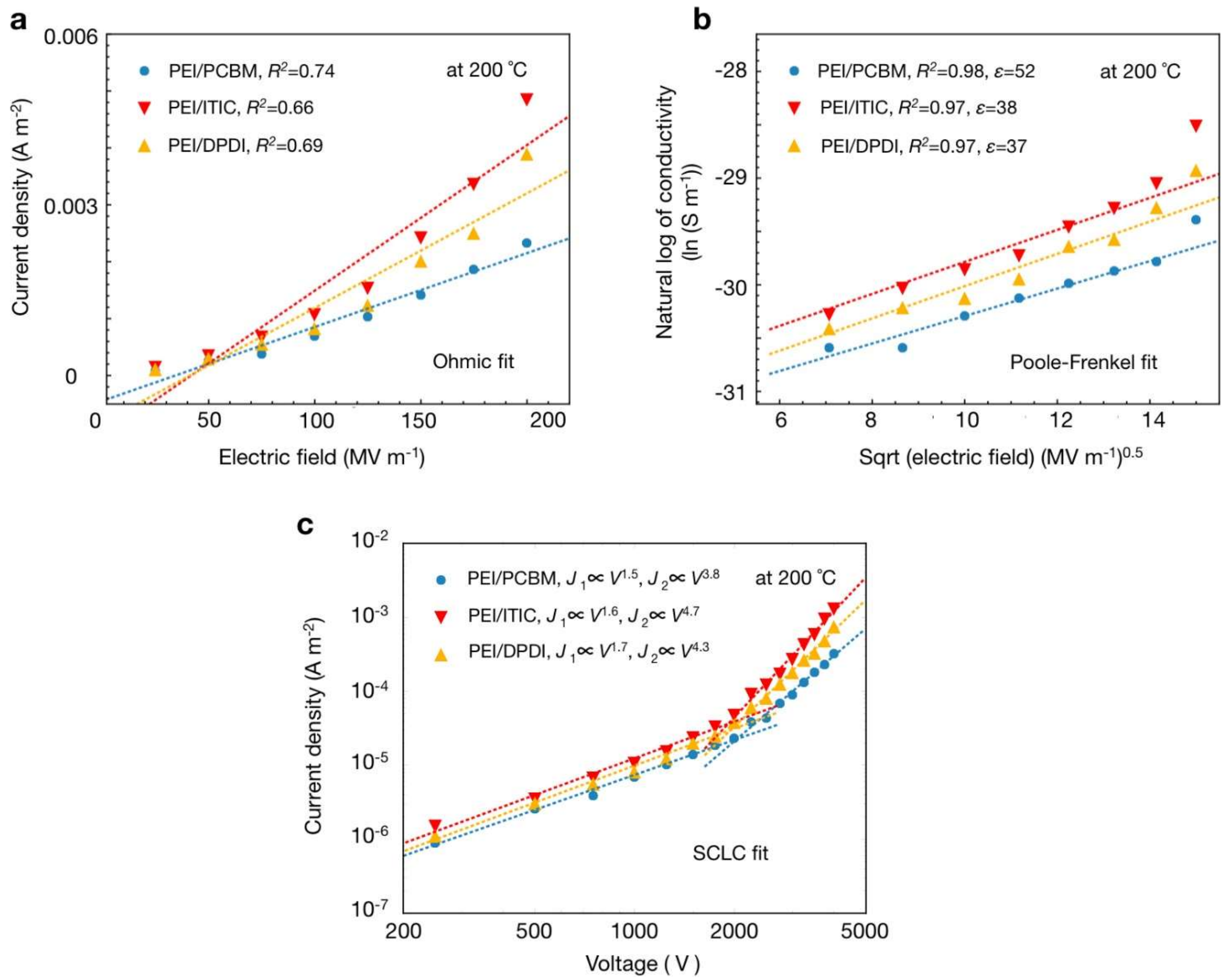

Supplementary Figure 17. Fittings to the (a) Ohmic conduction, (b) the Poole-Frenkel emission and (c) the SCLC models. The current densities and conductivities are all measured at 200 °C.

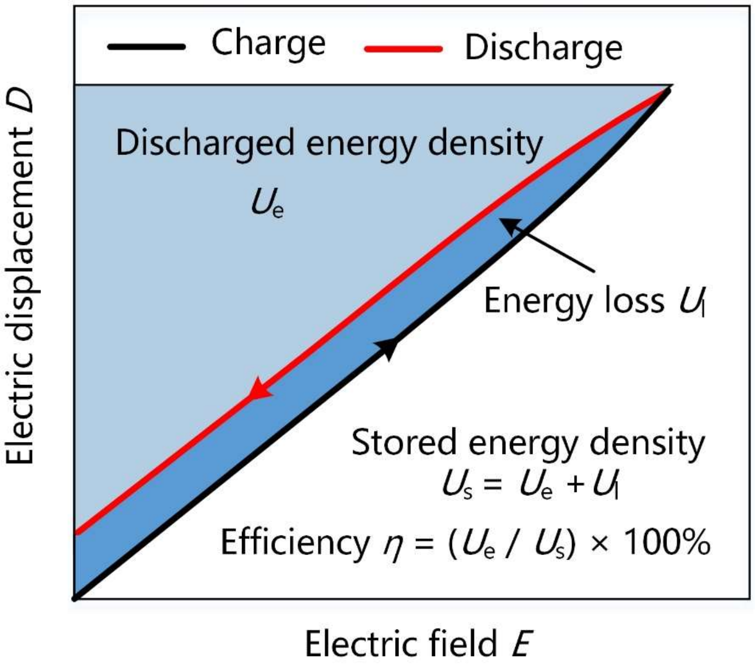

Supplementary Figure 18. Schematic unipolar *D-E* loop of a dielectric material. The discharged energy density (U_e) is represented by the area (colored in light blue) bounded by the line of discharge, the *D* axis and the horizontal line. The energy loss (U_l) is represented by the area (colored in dark blue) bounded by lines of charge and discharge, and the *D* axis. The stored energy density $U_s = U_e + U_l$. The discharge efficiency can be calculated by using the equation $\eta = (U_e / U_s) \times 100\%$.

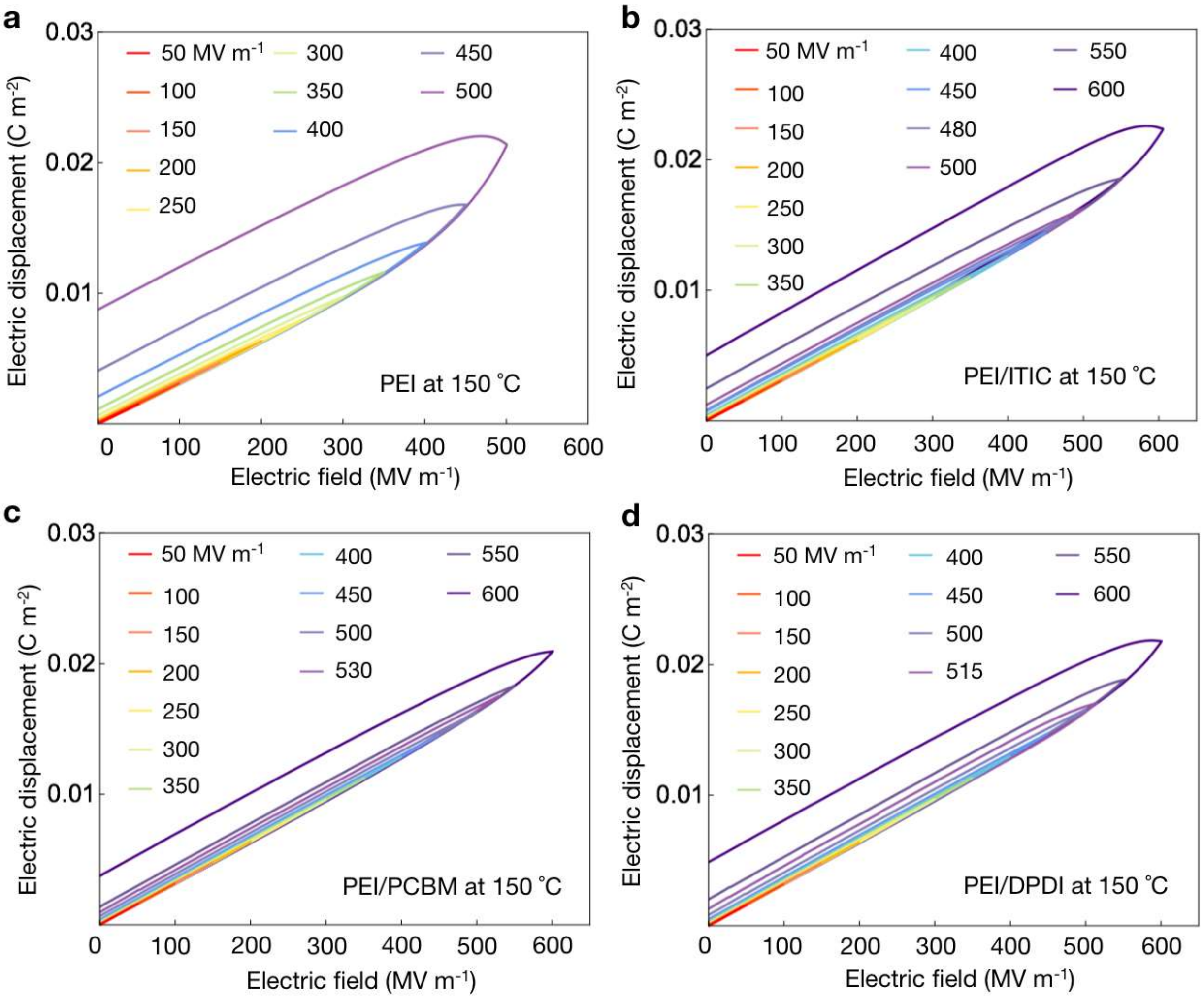

Supplementary Figure 19. Unipolar *D-E* loops of PEI (a), PEI/ITIC (0.25 vol.% ITIC) (b), PEI/PCBM (0.5 vol.% PCBM) (c) and PEI/DPDI (0.75 vol.% DPDI) (d) measured at 150 °C.

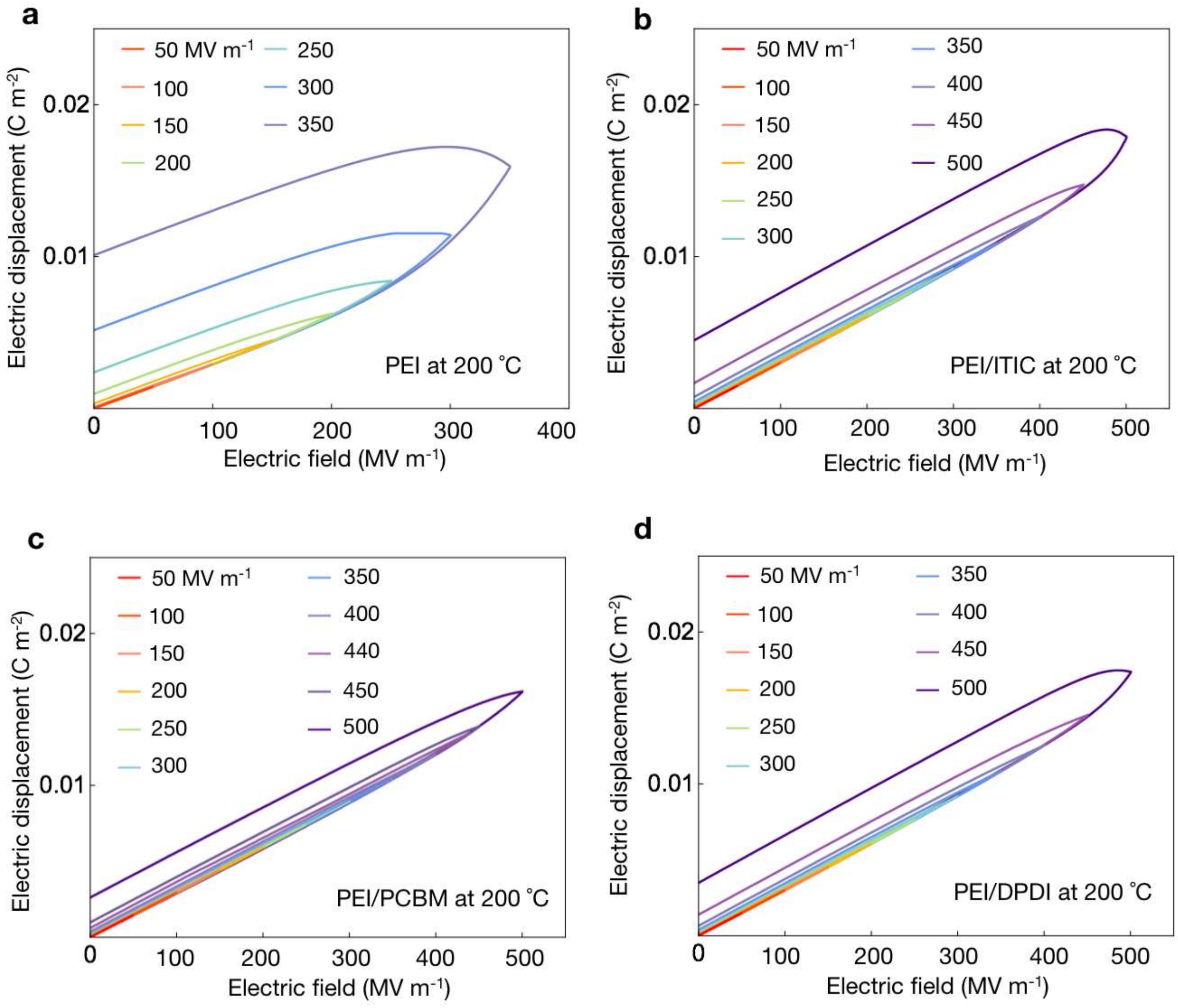

Supplementary Figure 20. Unipolar *D*-*E* loops of PEI (a), PEI/ITIC (0.25 vol.% ITIC) (b), PEI/PCBM (0.5 vol.% PCBM) (c) and PEI/DPDI (0.75 vol.% DPDI) (d) measured at 200 °C.

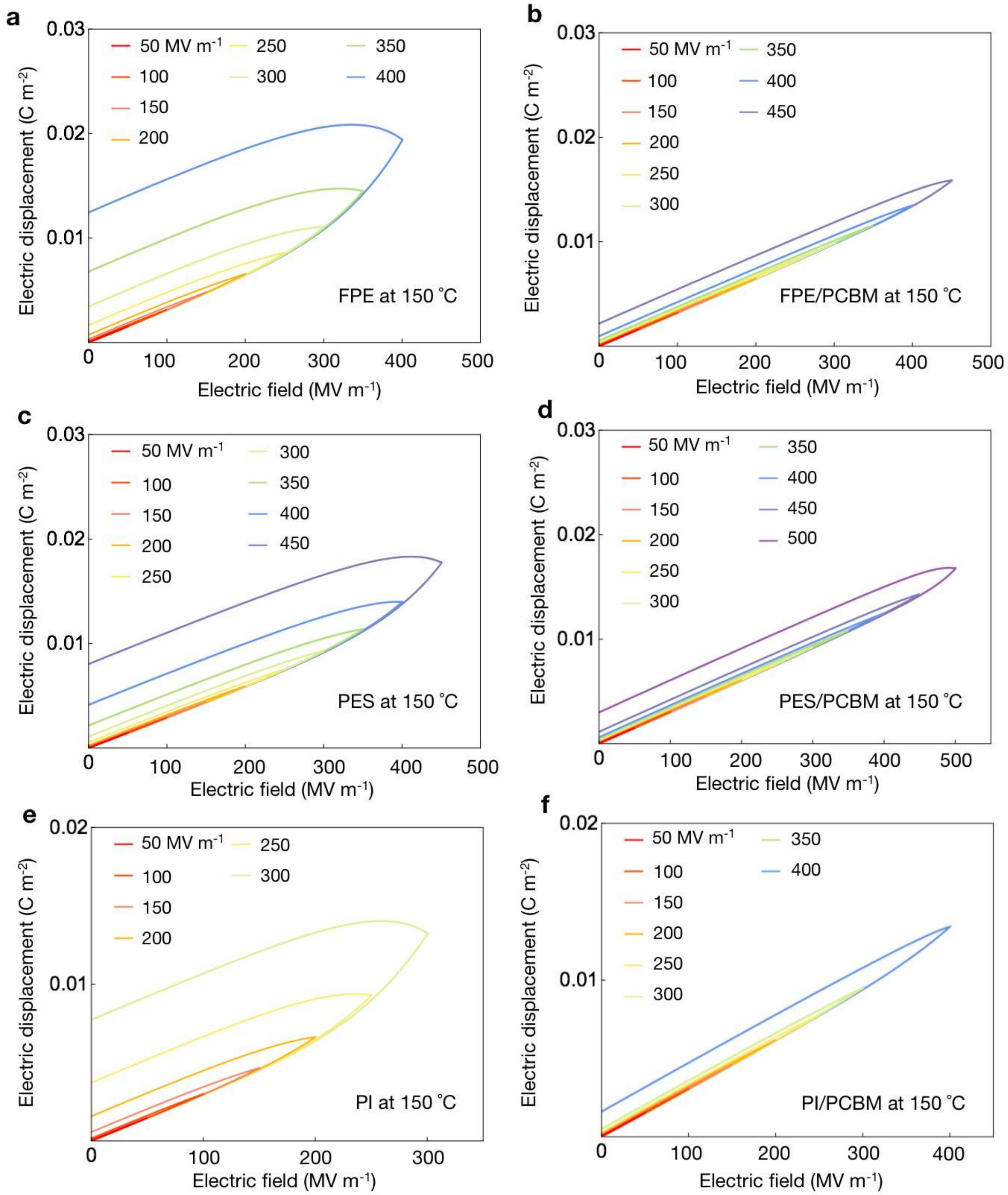

Supplementary Figure 21. Unipolar *D*-*E* loops of pristine FPE (a), FPE/PCBM (0.5 vol.% PCBM) (b), pristine PES (c), PES/PCBM (0.5 vol.% PCBM) (d), pristine PI (e) and PI/PCBM (0.5 vol.% PCBM) (f) measured at 150 °C.

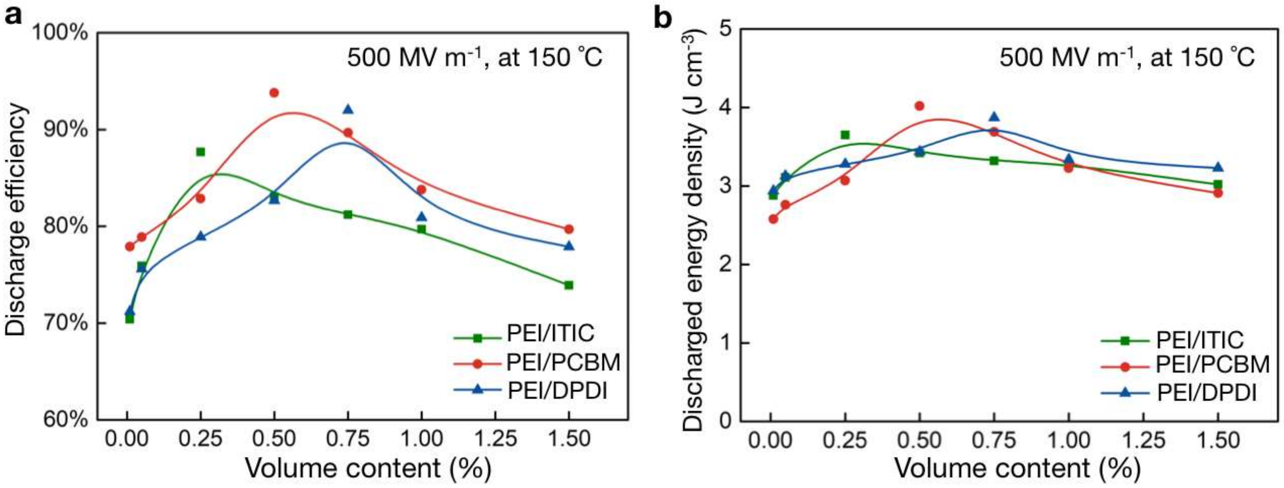

Supplementary Figure 22. Charge-discharge efficiency (a) and discharged energy density (b) of the PEI-based composites as a function of the ITIC, PCBM and DPDI content measured at 500 MV m^{-1} and 150 °C.

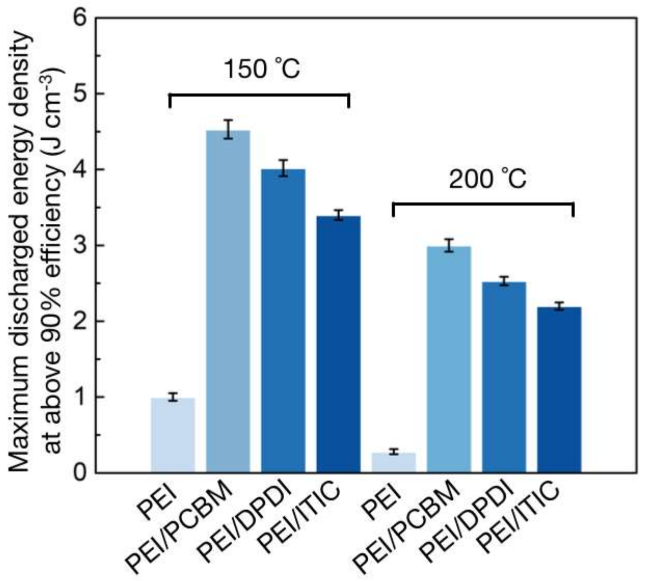

Supplementary Figure 23. Maximum discharged energy density above 90% efficiency of PEI and the all-organic composites at 150 °C and 200 °C. The average values and max–min error bars of the results were obtained from five parallel samples.

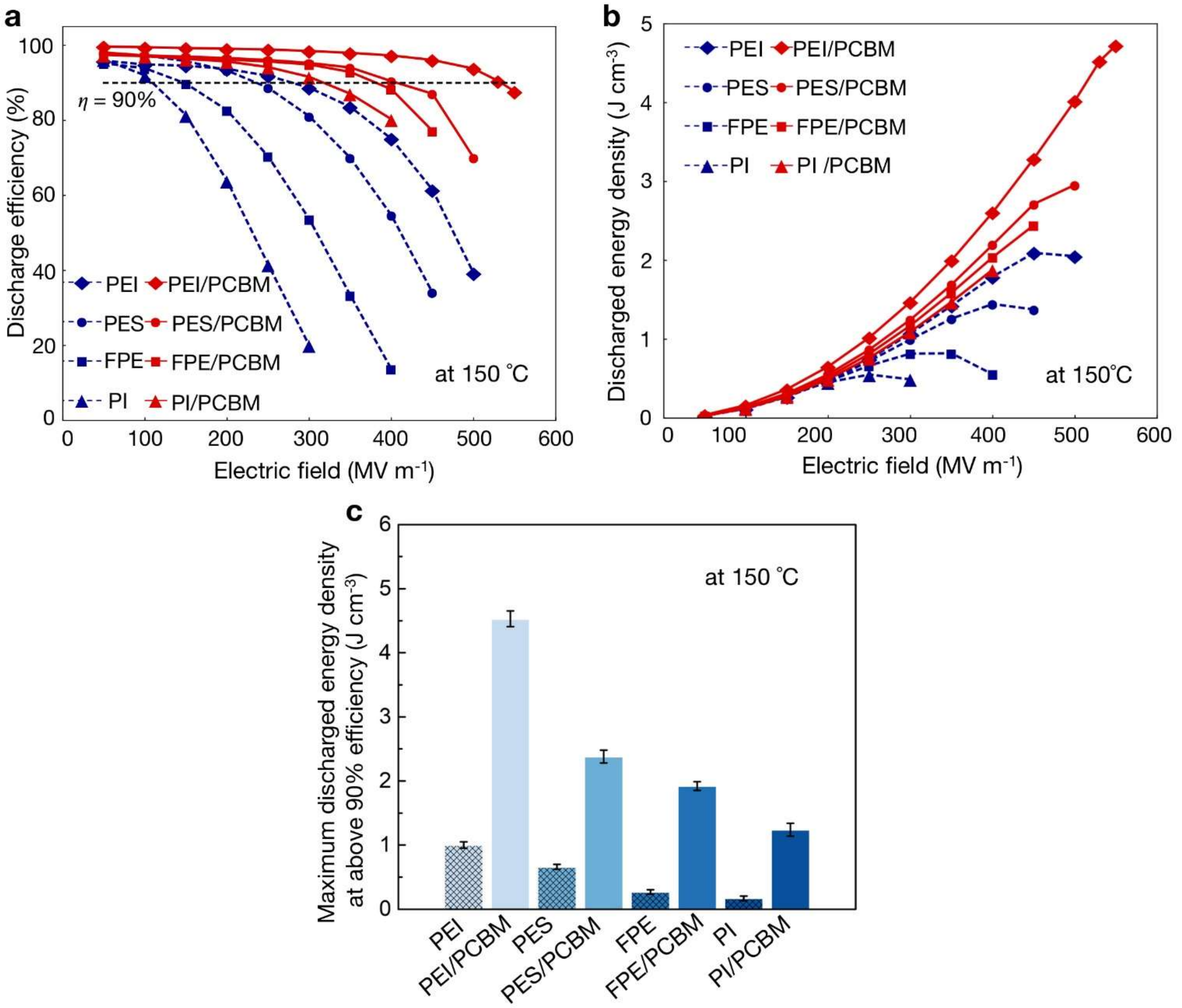

Supplementary Figure 24. (a) Field-dependent discharge efficiency and (b) discharged energy density, and (c) maximum discharged energy density above 90% efficiency of the various dielectric polymers incorporated with 0.5 vol.% PCBM at 150 °C. The average values and max–min error bars of the results were obtained from five parallel samples.

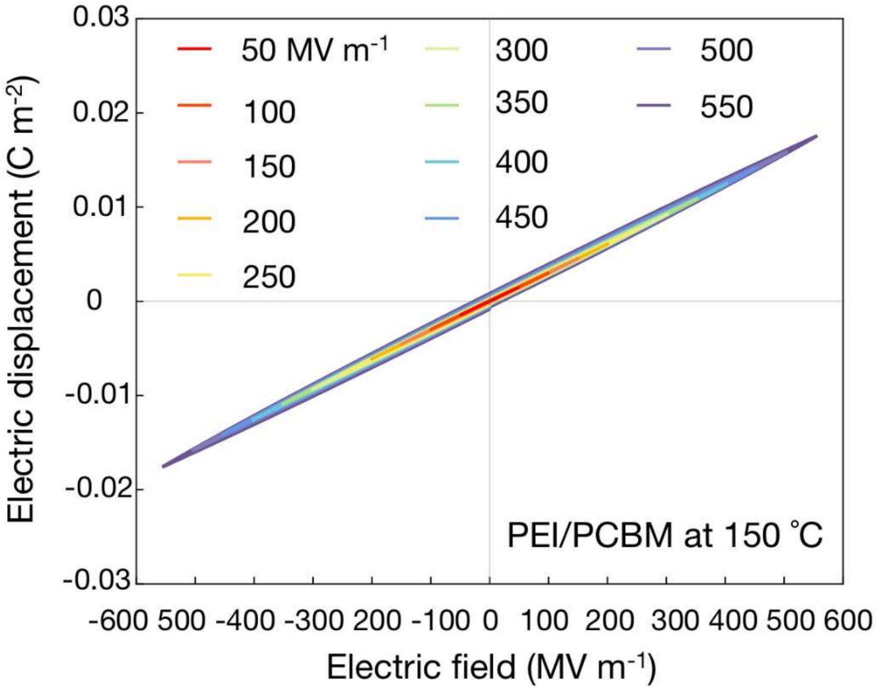

Supplementary Figure 25. Bipolar *D*-*E* loops of PEI/PCBM (0.5 vol.% PCBM).

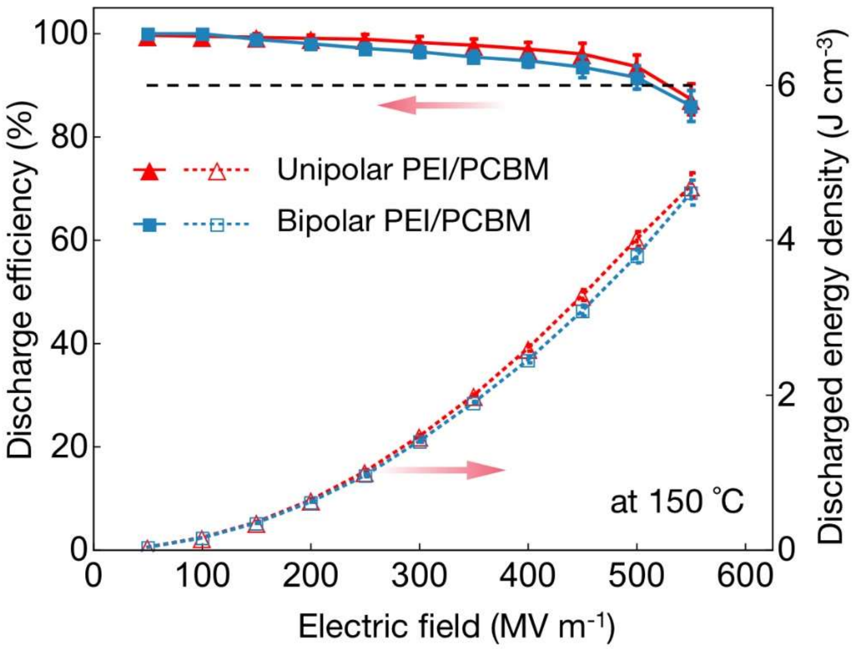

Supplementary Figure 26. Comparison of field-dependent discharge efficiency and discharged energy density of PEI/PCBM (0.5 vol.% PCBM) derived from unipolar and bipolar loops. The average values and max–min error bars of the results were obtained from five parallel samples.

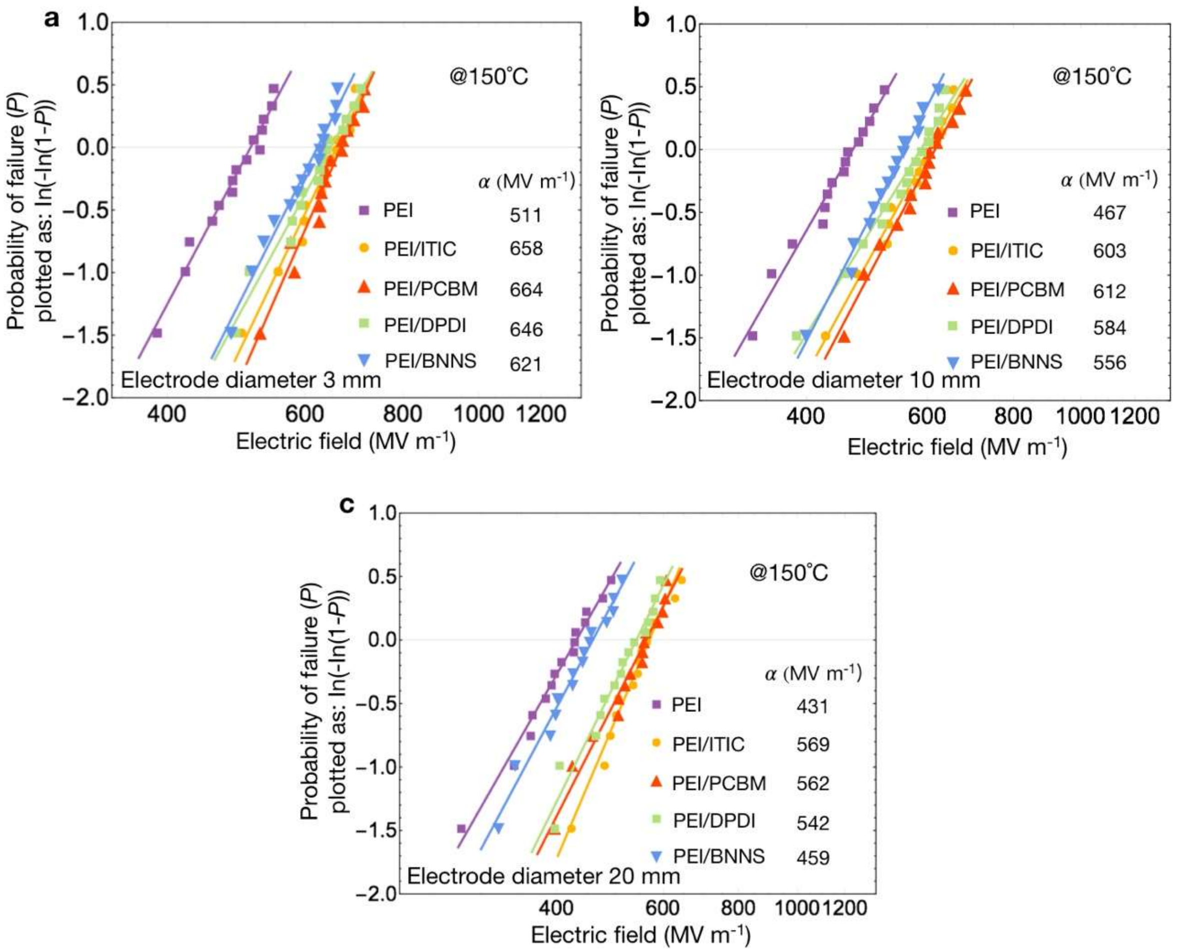

Supplementary Figure 27. Weibull distribution analysis of the breakdown strengths of PEI, PEI/ITIC (0.25 vol.% ITIC), PEI/PCBM (0.5 vol.% PCBM), PEI/DPDI (0.75 vol.% DPDI) and PEI/BNNS (10 vol.% BNNS) at 150 °C with (a) 3-mm-diameter electrodes, (b) 10-mm-diameter electrodes and (c) 20-mm-diameter electrodes.

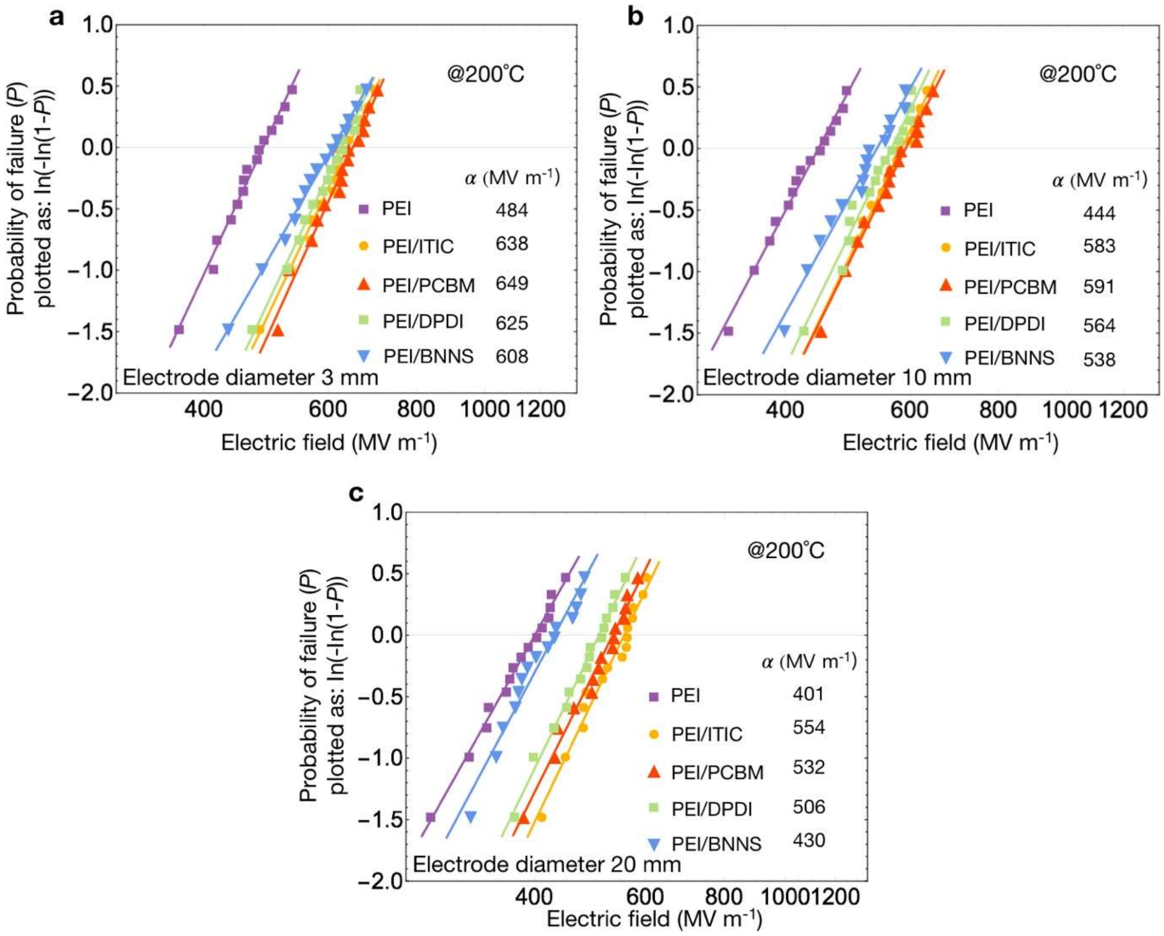

Supplementary Figure 28. Weibull distribution analysis of the breakdown strengths of PEI, PEI/ITIC (0.25 vol.% ITIC), PEI/PCBM (0.5 vol.% PCBM), PEI/DPDI (0.75 vol.% DPDI) and PEI/BNNS (10 vol.% BNNS) at 200 °C with (a) 3-mm-diameter electrodes, (b) 10-mm-diameter electrodes and (c) 20-mm-diameter electrodes.

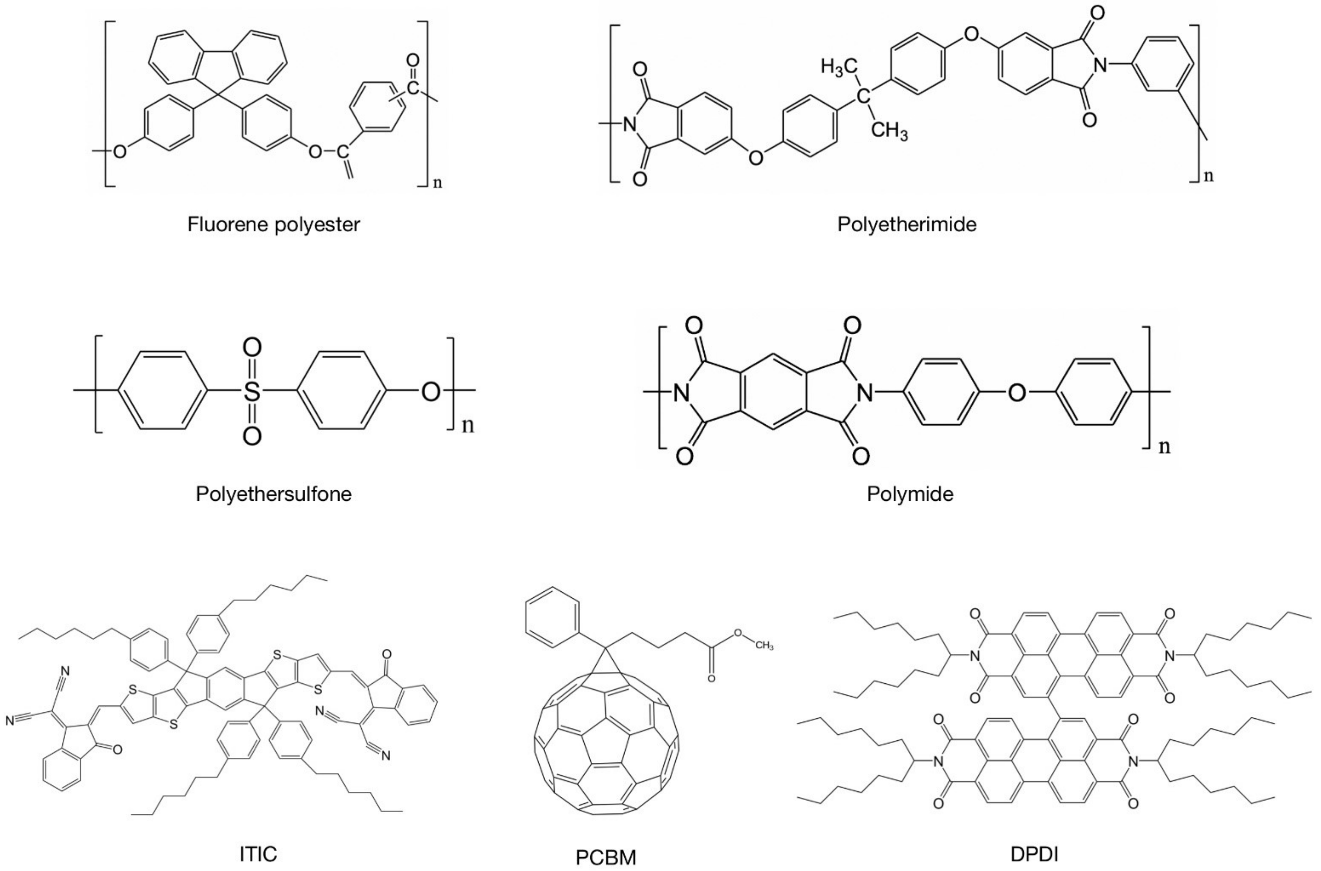

Supplementary Figure 29. Chemical structures of the dielectric polymers and the molecular semiconductors.

Supplementary Notes

Supplementary Note 1. Principle of KPFM measurement

A PeakForce KPFM approach is introduced to directly detect the variation of charges at local areas, and the working principle of the PeakForce KPFM is schematically shown in Supplementary Figure 11. The detection is implemented by a Bruker dimension icon scanning probe microscope. The conductive probe (PFQNE-AL) has a highly doped silicon tip without metal coating, which keeps the results stable and reduces the possible charge transfer between the probe and the sample.

The test is performed in two scanning steps. In the first scanning, the PeakForce Tapping mode[5] imaging is performed to obtain the topography of a scan line, which enables precise control over the contact force between the tip and the sample to reduce sample damage and increase resolution. In the second scanning, the topography information is used to retrace the baseline and the probe scans at a given lift height z (here, z is set to 50 nm) above the surface of sample. An external voltage $V = V_{\mathrm{ac}} \sin(\omega t)$ was applied to the tip (the driving frequency of AC voltage is 10 kHz, and the amplitude is 4 V), and the electric potential difference between the tip and the sample can be expressed as

$$V_{\mathrm{ts}} = V_{\mathrm{ac}} \sin(\omega t) - \Delta V \tag{1}$$

where $\Delta V = \phi_{\mathrm{sample}} - \phi_{\mathrm{tip}}$ represents the surface potential difference between the tip and the sample, which is related to the difference of work function ($\Delta\Phi$) between the tip and the sample and the charges in the sample μ, that is, $\Delta V = \Delta\Phi/e + \mu$ (Ref. 6). The electrostatic force on the tip can be written as

$$F = \frac{1}{2}\frac{\partial C}{\partial z} V_{\mathrm{ts}}^{\ 2} \tag{2}$$

substituting Supplementary Eq. 1 into Supplementary Eq. 2, and get the ω and 2ω components of electrostatic force:

$$F_{\omega} = \frac{\partial C}{\partial z} \Delta V \cdot V_{\mathrm{ac}} \sin(\omega t) \tag{3}$$

$$F_{2\omega} = \frac{1}{4}\frac{\partial C}{\partial z} V_{\mathrm{ac}}^{2} \cos(2\omega t) \tag{4}$$

where C is the capacitance between the probe/cantilever and the sample. Because van der Waals force is a kind of short-range force, while electrostatic force is a kind of long-range force, when the probe is lifted, van der Waals force decreases rapidly[7], the interaction force between the probe and sample is mainly the electrostatic force.

Due to the impact of electrostatic force, the vibration of the probe can be described as a forced Lorentz oscillator model[8]:

$$m\ddot{x} + \frac{m\omega_0}{Q}\dot{x} + kx = F_{\mathrm{d}} \cos \omega t \tag{5}$$

where m, Q and k are the mass, quality factor and elastic coefficient of the probe cantilever, respectively, x and ω_0 are the vibration displacement and resonance angular frequency of the probe cantilever, respectively, and $\omega_0 = \sqrt{k/m}$, F_{d} and ω are the amplitude and angular frequency of driving force applied on the tip, that is, the electrostatic force F. Since the van der Waals force is ignored, the driving force is mainly the electrostatic force. The vibration displacement of the probe cantilever can be solved as

$$x = Be^{-\alpha t}\cos\left(\omega_{\mathrm{r}} t + \beta\right) + A\cos\left(\omega t - \phi\right) \tag{6}$$

the steady-state solution is

$$x = A\cos\left(\omega t - \phi\right) \tag{7}$$

$$A = \frac{|F|}{m\sqrt{\left(\frac{\omega_0 \omega}{Q}\right)^2 + \left(\omega_0^2 - \omega^2\right)^2}} \tag{8}$$

when ω and 2ω is far less than ω_0, $A \propto |F|$, then we get

$$A_\omega \propto F_\omega = \frac{\partial C}{\partial z}|\Delta V| V_{\mathrm{ac}} \tag{9}$$

$$A_{2\omega} \propto F_{2\omega} = \frac{1}{4}\frac{\partial C}{\partial z} V_{\mathrm{ac}}^2 \tag{10}$$

$$\Delta V = \pm \frac{1}{4} V_{\mathrm{ac}} \frac{A_\omega}{A_{2\omega}} \tag{11}$$

As shown in Supplementary Eq. 11, ΔV can be calculated via V_{ac}, A_{ω} and $A_{2\omega}$, which can be easily obtained from the measurement.

Supplementary Note 2. Detection of charge injection

The detection of local surface potential (ΔV) at the electrode/dielectric interface was conducted to reveal the effect of molecular semiconductors on the injected charges (Fig. 2a in the main text). A customized substrate with a pair of aluminum electrodes (Supplementary Figure 12) was used to apply a DC voltage on the PEI/PCBM film and to inject charges prior to the KPFM measurement. The aluminum metal was selected as the electrode material because of its suitability for the fabrication process of electrodes (vacuum deposition followed by photolithography). The DC field strength was set at 0.5 MV m^{-1} to avoid breakdown of the air, and the DC field was held for 120 minutes and the sample temperature was set at 80 °C to allow for adequate charge injection. The KPFM measurement was performed 5 minutes after the DC field was removed, and there was no DC bias during the KPFM measurement. In addition to the test on the PEI/PCBM with charges being injected (the results are shown in Fig. 2 in the main text), we carried out two control experiments. The first one is that we did the same measurement on a pristine PEI sample (Supplementary Figure 14 a and b). The mapping of local surface potential on the pristine PEI shows a slight difference in contrast between the electrode and the polymer (Supplementary Figure 14b). This is mainly ascribed to the electrostatic charge injection effect and the different energy levels between the metal electrode (aluminum) and the dielectric (PEI). The second control experiment was implemented using the PEI/PCBM film but without applying the DC electric field prior to the KPFM measurement (Supplementary Figure 14 c and d). It was found that without the charge injection, the ΔV signal remained almost unchanged at the interface, which is very different from the result shown in Fig. 2c. Therefore, according to the test principle of KPFM described above, it is reasonable to conclude that the line signal at the interface in Fig. 2c is mainly due to the contribution of charge injection, rather than the difference in work function of the materials.

Supplementary Note 3. Conduction in the lower electric field regime

In the lower electric field regime discussed in Fig. 2f of the main text, we assign the Schottky emission the most plausible conduction mechanism. The Schottky emission[10] is a conduction mechanism usually working at high temperatures. In this case, carriers can overcome the energy barrier at the electrode/dielectric interface and be injected into the dielectric. As the electric field is increased, the barrier height is lowered due to the superposition of the image charge potential with the external potential. The Schottky emission based on leakage density (J) and electric field (E) can be expressed by

$$J = AT^2 \exp\left[-\left(\frac{\varphi - \sqrt{e^3/4\pi\varepsilon}\sqrt{E}}{K_B T}\right)\right] \tag{12}$$

where E is the electric field, A the Richardson constant, φ the barrier height at the electrode/dielectric interface, T the temperature, ε the dielectric constant, and K_B the Boltzmann constant. Supplementary Eq. 2 can be rewritten as

$$\ln\left(\frac{J}{T^2}\right) = \left(\frac{\sqrt{e^3/4\pi\varepsilon}}{K_B T}\right)\sqrt{E} + \ln(A) - \left(\frac{\varphi}{k_B T}\right) \tag{13}$$

From Supplementary Eq. 13, the plot of $\ln(J/T^2)$ versus$\sqrt{E}$ shall exhibit a linear relationship. The dielectric constant derived from the slope of fitted curve can be used to determine whether the conduction mechanism in the dielectric conforms to the Schottky emission. The dielectric constants derived for the PEI and PEI/BNNS are 3.3 and 4.1, respectively, very close to the experimental data, suggesting that these two materials are characteristic of the Schottky emission in the lower electric field regime. On the other hand, the dielectric constants derived for the all-organic composites range from 6.2 to 7.0, showing a large discrepancy from the experimental data. This result indicates that the all-organic composites do not follow a typical Schottky emission conduction model in the lower electric field regime. To this end, we tried to fit the data to other plausible conduction mechanisms, including the Ohmic conduction, the Poole-Frenkel emission and the space-charge-limited conduction (SCLC). The results are shown in Supplementary Figure 17. It is evident that none of these conduction mechanisms matches the experimental data. To be specific, the qualities of fitting, R_2, are too low to validate the Ohmic model. For the Poole-Frenkel fit, the estimated dielectric constants (ε) are too much higher than the experimental data, which excludes this conduction model. For the SCLC fit, the current density versus voltage (J versus V) curves are discrepant from the V^1 or V^2 exponents, indicating the conductions do not follow the ideal SCLC behavior. Therefore,

we speculate that the presence of the built-in field restricting the charge injection leads to the modified conduction mechanism in the lower electric field regime of the all-organic composites.

Supplementary Note 4. Conduction in the higher electric field regime

In the higher electric field regime discussed in Fig. 2f of the main text, we assign the hopping conduction the most plausible mechanism. The hopping conduction is a bulk mechanism that models the movement of individual carriers, assumed to be electrons, through the material. The carriers gain energy through random thermal fluctuations and phonon interaction to escape their localized state and travel in an extended state for a small amount of time before being recaptured by another localized state.

$$J = n_{\mathrm{c}} 2\upsilon\lambda e * \exp\left(-\frac{W_{\mathrm{a}}}{K_{\mathrm{B}}T}\right)\sinh\left(\frac{\lambda eE}{2K_{\mathrm{B}}T}\right) \tag{14}$$

where n_c is the carrier concentration, λ the hopping distance, v the attempt-to-escape frequency, W_a the activation energy in eV, e the charge of the carriers, T the temperature, K_B the Boltzmann constant. Fitting the *J–E* data to the hopping conduction model gives the hopping distance λ (i.e., mean spacing between trap sites). From Supplementary Eq. 14, the plot of J versus E shall exhibit a hyperbolic sine relationship. The hopping distance λ can be derived from the fitted curves, which are 1.84 nm, 1.67 nm, 1.24 nm, 1.1 nm and 0.89 nm for the pristine PEI, PEI/BNNS, PEI/ITIC, PEI/DPDI and PEI/PCBM, respectively.

Supplementary Note 5. The Arrhenius function of conductivity

Activation energies can be determined from the high-field temperature dependent conductivities, which generally fit an Arrhenius equation given as,

$$\sigma(T) = \sigma_0 * \exp\left(-\frac{W_{\mathrm{a}}e}{K_{\mathrm{B}}T}\right) \tag{15}$$

where σ_0 is the prefactor, W_a is the activation energy in eV, e is the electronic charge, and K_B is the Boltzmann constant. From Supplementary Eq. 15, the plot of $ln\sigma$ versus $1/T$ shall exhibit a linear relationship. the average activation energies can be obtained from the slope of the fitted curve.

Supplementary Note 6. Two-parameter Weibull statistic

Dielectric breakdown behavior is analyzed with a two-parameter Weibull statistic described as

$$P(E) = 1 - \exp\left(-(E/\alpha)^{\beta}\right) \tag{16}$$

where $P(E)$ is the cumulative probability of electric failure, E is the measured breakdown field, the scale parameter α is defined as the characteristic breakdown strength for which there is a 63% probability of sample breakdown, the shape parameter β measures the slope of the fitted Weibull curve, representing the scattering of the experiment data.

Supplementary References

1. Liu, J., *et al.*, Metal-free efficient photocatalyst for stable visible water splitting via a two-electron pathway. *Science* **347**, 970-974 (2015).
2. Samira, F., Sharma, H. & Shankar, K. Interfacial band alignment for photocatalytic charge separation in TiO_2 nanotube arrays coated with CuPt nanoparticles. *Phys. Chem.* **17**, 29723-29733 (2015).
3. Wang, C., *et al.*, Charge transfer at the PTCDA/black phosphorus interface. *J. Phys. Chem. C* **121**, 18084-18094 (2017).
4. Tian, F., *et al.*, Theory of modified thermally stimulated current and direct determination of trap level distribution. *J. Elec.* **69**, 7-10 (2011).
5. Peng, S., *et al.*, Direct detection of local electric polarization in the interfacial region in ferroelectric polymer nanocomposites. *Adv. Mater.* **31**, 1807722 (2019).
6. Hutchison, J. A., *et al.*, Tuning the work-function via strong coupling. *Adv. Mater.* **25**, 2481-2485 (2013).
7. Saint, M. J., Hudlet, S., Guthmann, C. & Berger, J. Van der Waals and capacitive forces in atomic force microscopies. *J. Appl. Phys.* **86**, 5245-5248 (1999).
8. Garcıa, R., Perez, R. Dynamic atomic force microscopy methods. *Sci. Rep*. **47**, 197-301 (2002).
9. Li, Y., Yasuda M. & Takada, T. Pulsed electroacoustic method for measurement of charge accumulation in solid dielectrics, *IEEE Trans. Dielectr. Electr. Insul.*, **1**, 188-195 (1994).
10. Barth, S., *et al.*, Current injection from a metal to a disordered hopping system. III. Comparison between experiment and Monte Carlo simulation. *Phys. Rev.* **60**, 8791 (1999).
